# Activity of Novel Synthetic Peptides against *Candida albicans*

**DOI:** 10.1038/srep09657

**Published:** 2015-05-12

**Authors:** Kah Yean Lum, Sun Tee Tay, Cheng Foh Le, Vannajan Sanghiran Lee, Nadia Hanim Sabri, Rukumani Devi Velayuthan, Hamimah Hassan, Shamala Devi Sekaran

**Affiliations:** 1 Department of Medical Microbiology, Faculty of Medicine, University of Malaya, Kuala Lumpur, Malaysia; 2 School of Pharmacy, Faculty of Science, University Nottingham Malaysia Campus, Semenyih, Selangor, Malaysia; 3 Department of Chemistry, Faculty of Science, University of Malaya, Kuala Lumpur

## Abstract

*Candida* spp. are the most common causes of fungal infections worldwide. Among the *Candida* species, *Candida albicans* remains the predominant species that causes invasive candidiasis in most countries. In this study, we used two peptides, KABT-AMP and uperin 3.6 as templates to develop novel antifungal peptides. Their anticandidal activity was assessed using a combination of MIC, time-killing assay and biofilm reduction assay. Hybrid peptides, KU2 and KU3 containing a mixed backbone of KABT-AMP and Uperin 3.6 demonstrated the most potent anticandidal activity with MIC values ranging from 8–16 mg/L. The number of Trp residues and the amphipathic structure of peptides probably enhanced the anticandidal activity of peptides. Increasing the cationicity of the uperin 3.6 analogues resulted in reduced MIC from the range of 64–128 mg/L to 16–64 mg/L and this was also correlated with the antibiofilm activity and killing kinetics of the peptides. Peptides showed synergistic effects when used in combination with conventional antifungals. Peptides demonstrated low haemolytic activity but significant toxicity on two normal human epithelial cell lines. This study provides us with a better understanding on the structure-activity relationship and the balance between cationicity and hydrophobicity of the peptides although the therapeutic application of the peptides is limited.

C*andida* spp. are human commensal microbes that commonly reside on skin, gastrointestinal tract, genitouninary system, oropharynx and upper respiratory tract without causing harm to healthy individuals^1^. However, when the host immune and defense system are debilitated, it becomes pathogenic and causes several types of infection, ranging from superficial infection, such as vulvovaginal candidiasis, esophageal or oropharyngeal candidiasis, to life-threatening invasive disorders including candidemia as well as disseminated and deep tissue candidiasis^1^,^2^.

*Candida* spp. are the most common causes of fungal infections worldwide. According to data reported by National Healthcare Safety Network (NHSN) at Centers of Diseases Control and Prevention (CDC), *Candida* spp. ranked the fifth among hospital-acquired pathogens^3^. In the United States, *Candida* spp. were the fourth most common causative pathogens of nosocomial bloodstream infection and associated with high morbidity and mortality rates^4^. Among 15 pathogenic *Candida* isolates, *Candida albicans* remains as the predominant species that causes invasive candidiasis in most countries. However, recent surveillance studies have demonstrated an increasing incidence of candidemia due to non-*albicans* species such as *C. glabrata*, *C. parapsilosis, C. tropicalis*, and *C. krusei*^4^,^5^,^6^,^7^,^8^.

The risk factors for invasive candidiasis include immune disorders, exposure to broad-spectrum antimicrobial agents, use of immunosuppressive drugs, solid organ or bone marrow transplantation, malignancies, parenteral alimentation and indwelling catheters^1^,^9^. Besides that, studies showed that critically ill patients in ICU with prolonged stay of more than 7 days are predisposed to candidemia and candiduria^5^,^10^.

Global report on antimicrobial resistance surveillance published by WHO documented that only three classes of conventional antifungal drugs are currently being used to treat invasive candidiasis; they include the azoles (i.e. fluconazole), the echinocandins (i.e. caspofungin) and the polyenes (i.e. amphotericin B)^11^. In recent years, antifungal resistances in *Candida* spp., especially in non-*albicans* species, have been observed. For example, *C. krusei* had reduced susceptibility to fluconazole and *C. glabrata* was resistant to both azoles and echinocandins^12^. The emergence of antifungal resistant pathogens has been of great concern and prompted the development of alternative antifungal agents.

Antimicrobial peptides (AMPs) are relatively small (generally 10 to 50 amino acids), cationic (+2 to +9), containing ≥30% hydrophobic residues and amphipathic (hydrophobic and hydrophilic amino acids on opposite faces) molecules which have been found in most living organisms ranging from bacteria to plants, invertebrate and vertebrate species^13^,^14^. They act as the first line of defense and encompass direct antimicrobial activity against a broad spectrum of invading pathogens including bacteria, fungi, viruses and protozoa by using different modes of action as described previously^15^,^16^. Thus, AMPs can be potentially promising candidates for development of novel therapeutic agents against pathogens.

Several approaches such as template-based design, biophysical modelling, and computer-aided design have been proposed to design synthetic AMPs^17^. Template-based strategy is known as *de novo* design of peptide analogues based on natural AMPs with known antimicrobial activity. This approach is often more convenient and has been extensively applied by researchers to elucidate the structure-activity relationship of peptides and further enhance the antimicrobial potency of natural AMPs with low toxicity to mammalian cells^18^,^19^. In our study, KABT-AMP, a synthetic peptide^20^ and uperin 3.6, a naturally occurring peptide^21^ with known activity were selected and modified in an attempt to improve their antifungal activity. Physicochemical properties including size, residue composition, overall charge, hydrophobicity, and amphipathic structure were taken into consideration while designing synthetic AMPs.

## Results

### Peptide design

**Figure 1:**
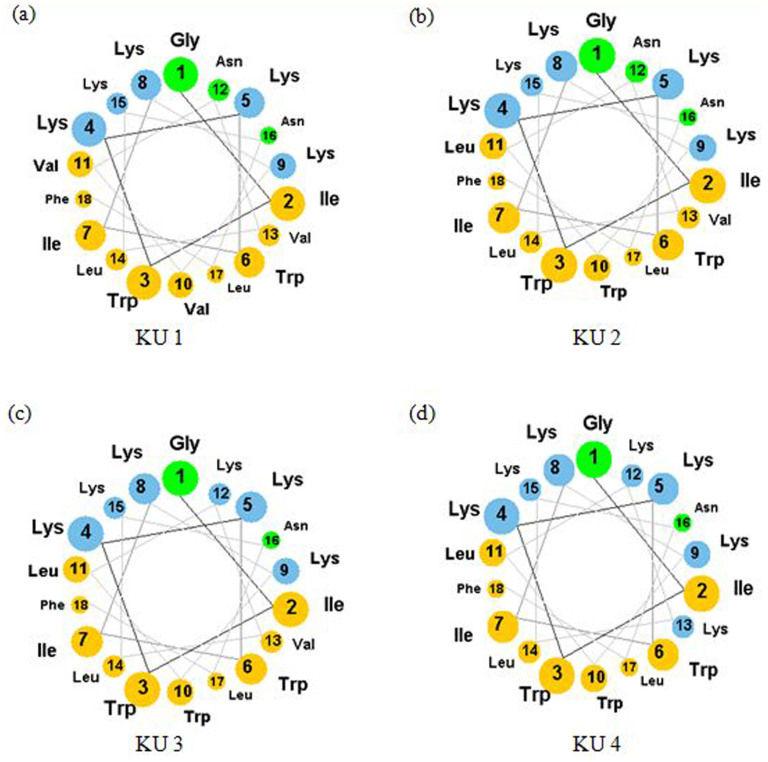
Helical wheel projections of (a) KU1, (b) KU2, (c) KU3 and (d) KU4. Yellow circles represent hydrophobic residues. Blue and green circles correspond to hydrophilic residues obtained from CAMP (Collection of Anti-Microbial Peptides) http://www.camp.bicnirrh.res.in/.

KABT-AMP and uperin 3.6 peptides were selected as templates for designing synthetic AMPs. Uperin 3.6 adopts a well-defined amphipathic α-helix, with hydrophobic side above and hydrophilic side below the polypeptide backbone. Synthetic modification performed by Chia *et al* had revealed that three lysine residues in the sequence of uperin 3.6 are crucial for antimicrobial activity^21^. To further enhance the antimicrobial activity of uperin 3.6, three lysine-substituted analogues of uperin 3.6 (Upn-lys4, Upn-lys5, and Upn-lys6) were designed by replacing less hydrophobic amino acids with cationic lysine residues so that activity variation depends solely on altering the charge. The overall charge of a parent peptide was increased to +4, +5 and +6 respectively without disrupting the amphipathic structure of the peptide. In addition, four hybrid peptides (KU1, KU2, KU3, and KU4) were designed by selecting and fusing peptide fragments from KABT-AMP and uperin 3.6. KU1 was designed by truncating the first nine residues at the N-terminus of KABT-AMP which was then fused to the 9^th^ to 17^th^ C-terminus of residual fragment (VVNVLKNLF-NH_2_) of uperin 3.6. Likewise, KU2, KU3 and KU4 were also designed using the same approach by truncating first eleven, twelve and thirteen residues at N-terminus of KABT-AMP, respectively, and fused with the respective 11^th^ to 17^th^, 12^th^ to 17^th^ and 13^th^ to 17^th^ C-terminus residues fragment of uperin 3.6 with amidation. The sequence and physicochemical properties of all designed peptides are provided in Table 1. The amphipathic orientation of the residues in helical structure of four hybrid peptides are displayed using the helical wheel projection (Figure 1).

### Anticandidal activity of designed peptides

The MIC of the reference and designed peptides against *C. albicans, C. krusei* and *C. parapsilosis* were determined as shown in Table 2. Conventional antifungal agents such as fluconazole and amphotericin B were included in this study as controls. Among the hybrid peptides, KU2 and KU3 demonstrated the most potent antifungal activity, showing MIC values ranging from 8 to 16 mg/L against four *Candida* strains. KU1 and KU4 also displayed good activity, with MIC values ranging from 16 to 32 mg/L and 16 to 64 mg/L respectively. All four KU hybrid peptides showed improved anticandidal activity when compared to their parent peptides KABT-AMP (MIC of 32 to 64 mg/L) and uperin 3.6 (MIC of 64 to 128 mg/L).

In terms of uperin 3.6 analogues, substitution of a single Lysine residue into the parent peptide (Upn-lys4) did not improve the antifungal activity against *C. albicans* strains and *C. krusei*. Upn-lys5 and Upn-lys6 displayed two-fold reduction in MIC values against *C. albicans* and *C. krusei* (MIC of 32 to 64 mg/L) suggesting that replacement of two lysine residues was required to improve the antimicrobial activity of the parent peptide by two-fold. With the exception of *C. parapsilosis*, the antimicrobial activity of uperin 3.6 was improved by twofold as the number of lysine residue substituted into template peptide increased. As for the standard antifungal drugs, amphotericin B has the most potent antimicrobial activity with MIC values ranging from 0.5 to 1 mg/L against *C. albicans* and non-*albicans* species. For fluconazole, two *C. albicans* strains and *C. parapsilosis* showed MIC values ranging from 1 to 2 mg/L while *C. krusei*, a species that is intrinsically resistant to fluconazole, demonstrated MIC value of 64 mg/L.

### Killing kinetics of designed peptides

**Figure 2:**
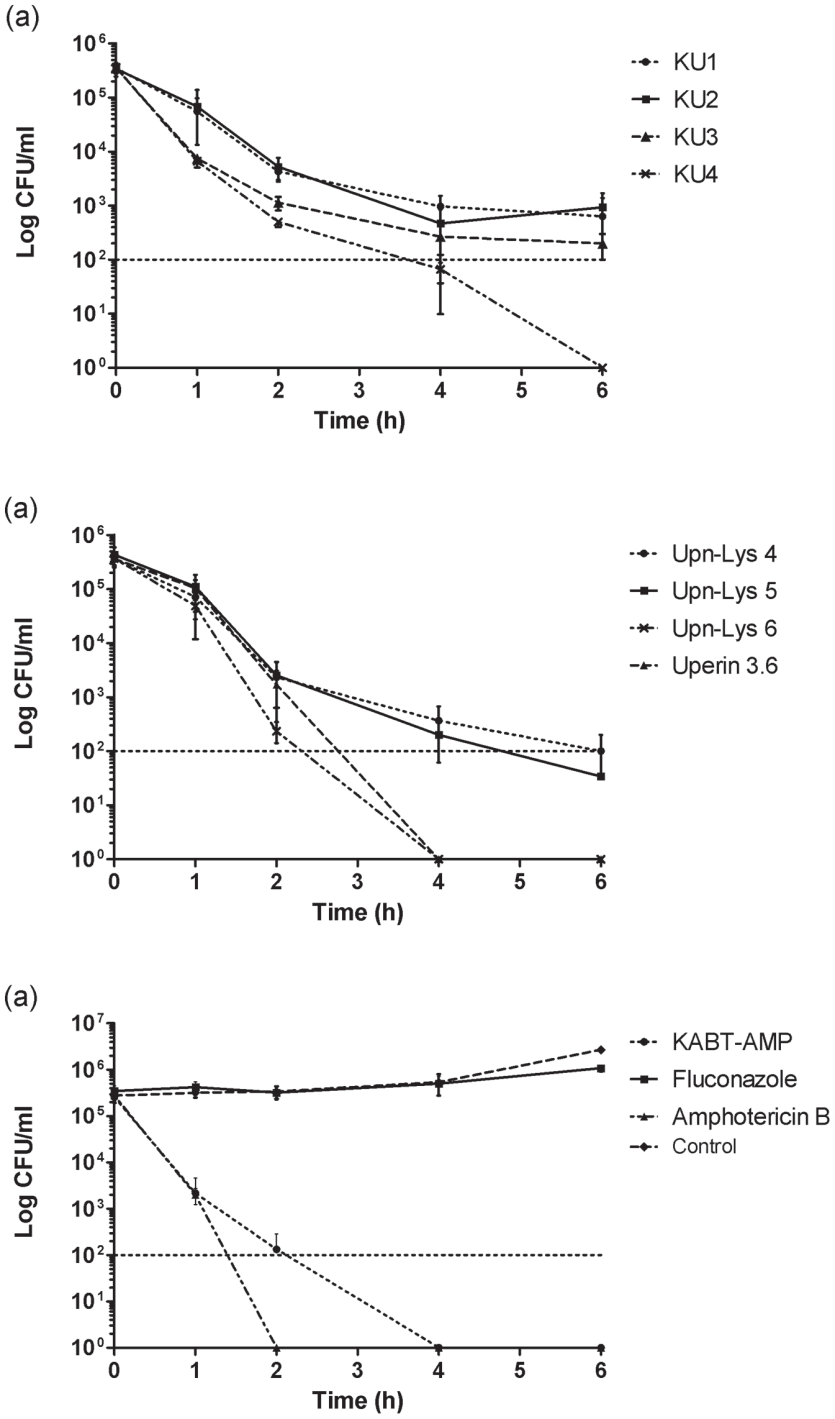
Killing kinetics of the designed peptides and antifungals against *C. albicans* SC 5314. Standardized yeast cells suspensions were exposed to a final concentration of two times the previously determined MIC of the tested strain. At determined time intervals, samples were serially diluted and plated for colony counts. Each data point represents mean result ± standard deviation (error bars) from three experiments.

Antifungal activity of the designed peptides was further assessed by determining their killing kinetics on *C. albicans* SC5314 strain at concentrations of 2× the respective MICs. Among the hybrid peptides, KU4 killed the yeast cells effectively, achieving a mean maximum log decrease of 5.55 ± 0.08 CFU/mL (>99.99% killing) within 6 hours of test period. KU1, KU2 and KU3 decreased the number of viable fungal cells with mean log reduction of 2.95 ± 0.59 CFU/mL (99.82% killing), 2.77 ± 0.54 CFU/mL (99.73% killing) and 3.28 ± 0.30 CFU/mL (99.94% killing), respectively, within 6 hours (Figure 2A). However, the parent peptide KABT-AMP caused 5.4 ± 0.09 log decreases in CFU/mL (>99.99% killing) within 4 hours, obtaining a better killing kinetics compared with the hybrid peptides (Figure 2C). In terms of uperin 3.6 analogues, Upn-Lys 6 and the parent peptide uperin 3.6 killed the yeast cells rapidly within 4 hours test period, exhibiting a mean maximum log reduction of 5.56 ± 0.07 CFU/mL (>99.99% killing) and 5.54 ± 0.13 CFU/mL (>99.99% killing), respectively. Upn-Lys 4 and Upn-Lys 5 attained respective log decrease of 4.12 ± 1.36 CFU/mL (99.97% killing) and 4.95 ± 1.24 CFU/mL (99.99% killing) within 6 hours (Figure 2B). With regard to the antifungal drugs, amphotericin B demonstrated the best killing kinetics, by eradicating the fungal cells rapidly within 2 hours with 5.44 ± 0.07 log reduction (>99.99% killing) in CFU/mL (Figure 2C). Fluconazole displayed a slight reduction in yeast growth (>99.9% reduction), showing fungistatic activity against the tested strain (Figure 2C).

### Biofilm reduction assay

**Figure 3:**
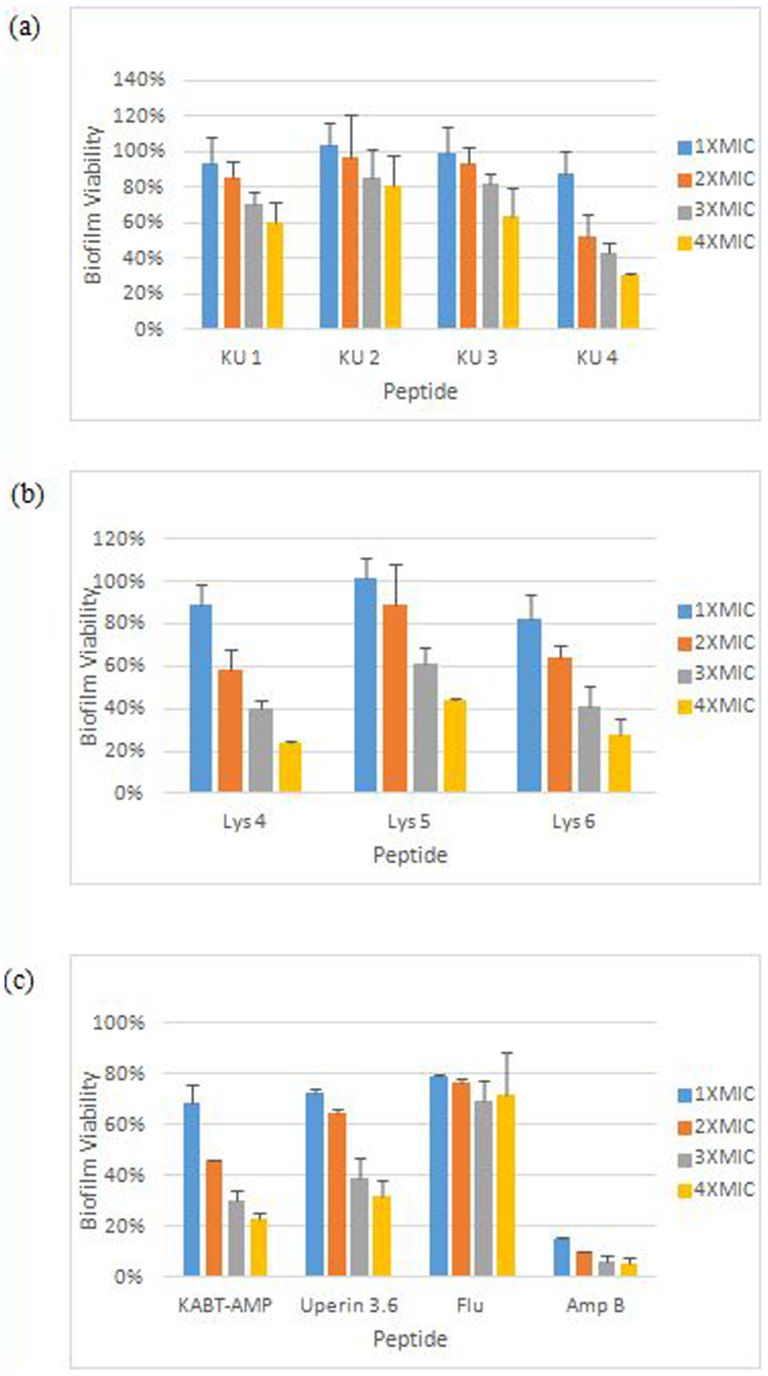
Biofilm viability after treatment with peptides at concentration ranging from 1 × to 4 × MIC against *C. albicans* SC5314 strain. Each data point represents mean result ± standard deviation (error bars) from two experiments in triplicates.

Biofilms formation is often associated with antifungal resistance as compared to planktonic cells and requires drug concentrations of 30–2000 times the corresponding MIC values to reduce 50% of the biofilms metabolic activity^22^. The susceptibility of the fungal biofilms to the designed peptides was assessed by using XTT reduction assay, which enables quantitation of the number of living cells in 24 hour-old biofilms after treatment. Based on the results shown in Figure 3, the biofilm metabolic activity of which Candida decreased as the concentration of peptides increased from 1× to 4× the planktonic MIC. KU4 with a BEC-2 value of 96 mg/L demonstrated the highest ability to eradicate biofilms among the hybrid peptides. The BEC-2 value for uperin 3.6 was 192 mg/L while its analogues, upn-lys4, upn-lys5 and upn-lys6 showed BEC-2 values of 192 mg/L, 128 mg/L and 96 mg/L, respectively. The parent peptide KABT-AMP exhibited good antibiofilm activity with a BEC-2 value of 64 mg/L. In contrast, the conventional antifungal agent, amphotericin B, displayed the most potent antibiofilm activity with a BEC-2 value of >1 mg/L while fluconazole had a BEC-2 value of >4 mg/L (>4 × MIC) (Table 3).

### Growth inhibitory effect of the peptide-peptide and peptide-antifungals combination

The *in vitro* synergism effects of the peptide-peptide and peptide-antifungals combination were identified by using a modified checkerboard method. Most of the peptide-peptide combinations showed no synergistic effect against *C. albicans* SC5314 and ATCC 90028 strains and were in the additive or indifferent range (Supplementary data: Table S1). All the peptide-amphotericin B combinations demonstrated synergistic effects with FIC indices of less than 0.5 when tested against *C. albicans* SC5314 strain. In contrast, there are only four peptide-fluconazole combinations (KU4, KABT-AMP, Upn-lys6 and uperin 3.6) that displayed synergistic effects against SC5314 strain. However, most of the peptides inhibited the growth of ATCC 90028 strain when tested in combination with amphotericin B except Upn-lys6 and uperin 3.6. In term of fluconazole, KU4 was the only peptide displaying a synergistic effect when used in combination with fluconazole against both strains.

### Cytotoxicity of peptides on normal human cells

Haemolytic assay was carried out to determine the ability of the peptides to disrupt the membrane integrity of mammalian cells. The hybrid peptide KU1 showed haemolytic activity (>10%) at concentrations lower than the MIC doses. KU2 and KU3 also displayed moderate haemolytic activity with HC_10_ and HC_50_ of 7.33 ± 0.73 mg/L and 57.71 ± 9.80 mg/L for KU2 and 5.65 ± 0.85 mg/L and 55.20 ± 11.90 mg/L for KU3. KU4 was the hybrid peptide with the least haemolytic activity (HC_10_ = 49.38 ± 5.14 mg/L and HC _50_ > 256 mg/L). Neither uperin 3.6 nor its analogs exhibited toxicity to human red blood cells (HC_10_ and HC_50_ of more than 256 mg/L) (Table 4).

The cytotoxicity of the designed peptides against human vaginal (VK2/E6E7) and esophagus (Het-1A) epithelial cell lines was assessed after 24, 48 and 72 hours of treatment by using MTS assay (Table 5). Significant toxic effects of the hybrid peptides and uperin 3.6 analogs were observed against VK2/E6E7 and Het-1A cell lines. Hybrid peptides and uperin 3.6 analogs reduced the cell viability to 50% at concentrations below the MIC doses. Uperin 3.6 and its analogs were highly toxic to both cell lines with low IC_50_ values although they did not exert haemolytic activity on human erythrocytes.

## Molecular interaction of designed peptides with SAP1, SAP 5 and exo-beta-(1, 3)-glucanase

Results obtained from rigid docking performed by Autodock Vina software indicated that all peptides bound to three selected target proteins respectively were within the negative binding affinity range (Supplementary data: Table S2). Peptides with greater negative binding affinity range indicate stronger binding affinities with target proteins. In comparison with peptides in respective series, KU3 and Upn-lys5 with lower MIC values showed higher binding affinities for three target proteins and hence, they were selected for further analysis. Parent peptide KABT-AMP and uperin 3.6 were included as a control (Supplementary data: Table S2).

**Figure 4:**
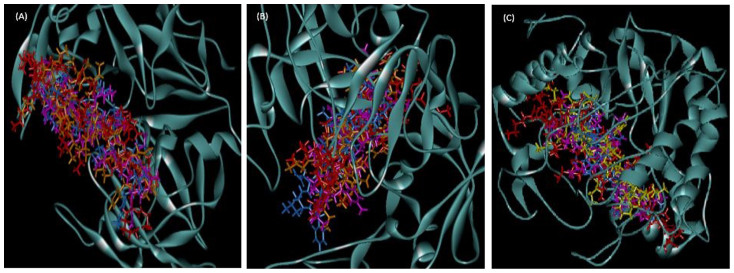
The superposition of peptide structure of KABT-AMP (red), KU3 (orange), uperin3.6 (blue) and Upn-lys5 (purple) in (A) sap1, (B) sap5 and (C) β-1,3-exoglucanase docking complexes.

**Figure 5:**
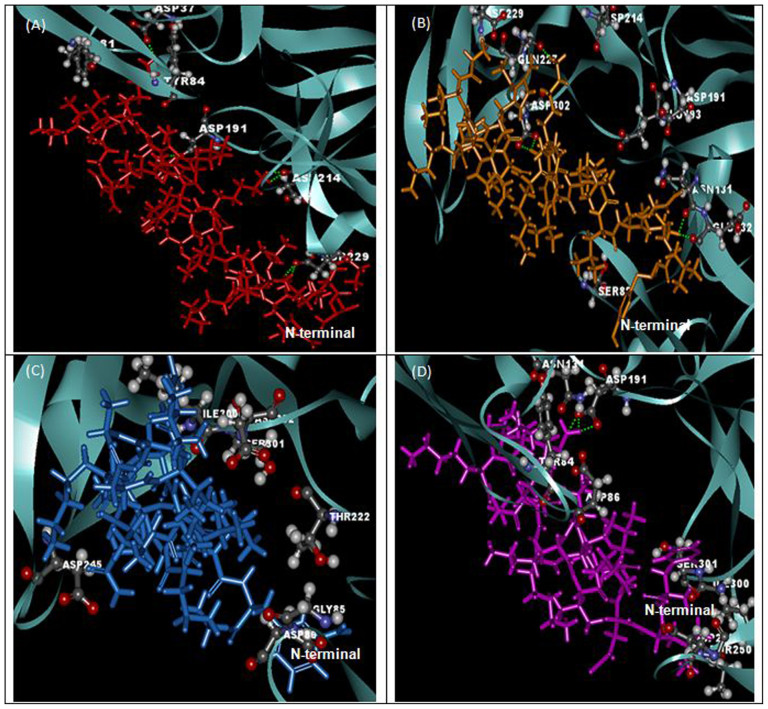
The docking conformation and sap1 amino acid interactions with (A) KABT-AMP, (B) KU3, (C) uperin3.6 and (D) Upn-lys5.

**Figure 6:**
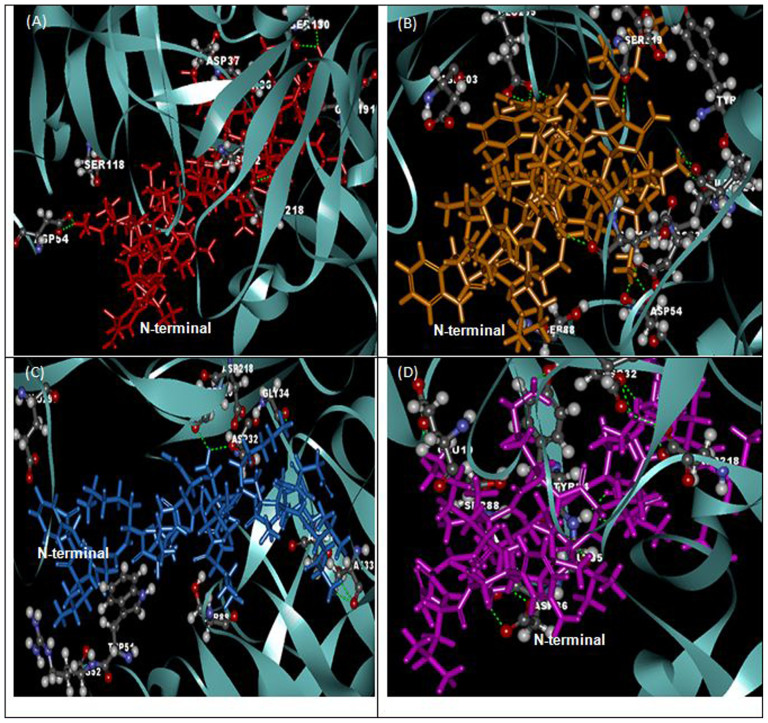
The docking conformation and sap5 amino acid interactions with (A) KABT-AMP, (B) KU3, (C) uperin3.6 and (D) Upn-lys5.

**Figure 7:**
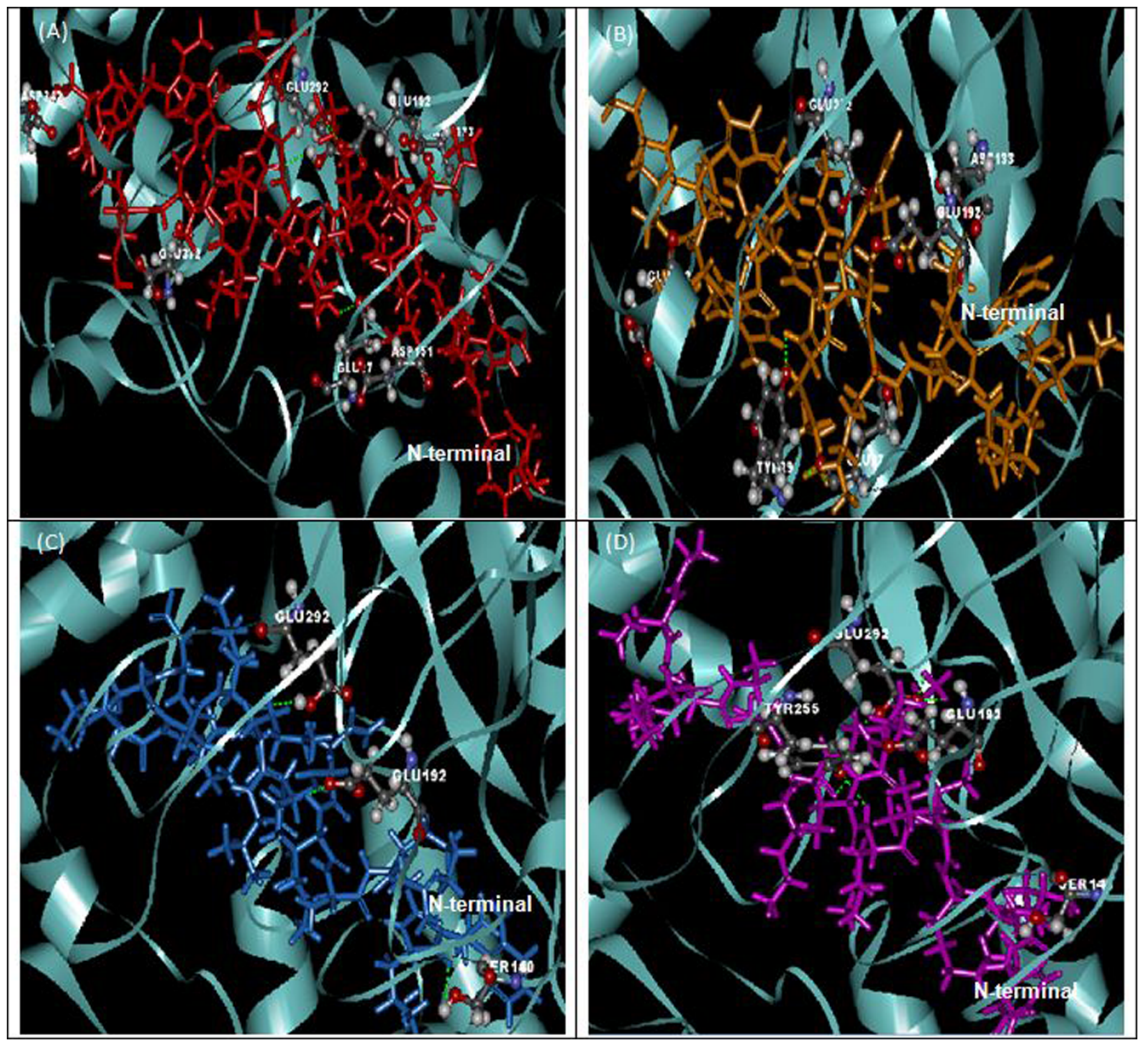
The docking conformation and β-1,3-exoglucanase amino acid interactions with (A) KABT-AMP, (B) KU3, (C) uperin3.6 and (D) Upn-lys5.

The superpositions of minimized docking structures for the peptides were shown in Figure 4. The docking conformation and the interaction between potential target proteins (sap1, sap5 and exo-β-(1, 3)-glucanases (Exg)) with peptides are shown in Figure 5, 6 and 7. The green dotted line represents hydrogen bonds of the complexes. Based on the docking conformation, KU3 and KABT-AMP displayed similar number of hydrogen bonds with sap1 (Figure 5). Docking conformation of KU3 showed hydrogen bonding between sap5 at Tyr14, Ile30, Asp37, Asp54, Lue124, Glu132, Asp132 and Glu295 residues (Figure 6). For Exg, hydrogen bonds formed between KU3 at Glu27, Tyr29 and Glu372 residues (Figure 7). On the other hand, Upn-lys5 formed more hydrogen bonds with the target proteins than Uperin3.6. Three hydrogen bonds formed between Upn-lys5 and sap1 at Asp191 whilst none was noted for Uperin3.6 (Figure 5). Upn-lys5 formed hydrogen bonds with sap5 at Glu10, Asp32, Tyr84, Gly85, Asp86 and Asp218 residues, as seen in Figure 6.

The total interaction energy of the target proteins and peptides, which is the sum of the van der Waals (VDW) and electrostatic energy within 3 Å, is tabulated (Supplementary data: Table S3, S4 and S5). KU3 shows strong binding interactions with the target proteins as compared to other peptides. For sap1, KABT-AMP showed slightly stronger interaction than KU3 mainly due to the electrostatic interaction with Asp37, Asp191 and ASP214. KU3 was found to exhibit interaction with Glu10, Asp32, Asp37, Asp54, Asp86, Glu295 and Asp308 of sap5 while for Exg, KU3 has interactions with Asp145, Asn191, Glu192, Gly291, Gly292, Asn299 and Asp318 residues. All peptides demonstrated interactions with Glu192 and Glu292 of Exg since both residues are catalytic sites. From the result, Upn-lys5 showed higher total interaction energy than Uperin3.6 for sap1 (−453.603 kcal/mol), sap5 (−1004.134 kcal/mol) and Exg (−627.185 kcal/mol).

## Discussion

The increased incidence of invasive fungal infections and the emergence of antifungal drug resistance in *Candida* spp., particularly against echinocandins and azoles, have prompted the development of alternative antifungal agents^12^. Antimicrobial peptides (AMPs) can be promising candidates for novel antifungal agents due to their broad-spectrum activities against pathogens and low chances of developing antimicrobial resistance^23^. Some naturally occurring antifungal peptides such as polymyxin, melittin and protegrin demonstrated potent antimicrobial activity, but their therapeutic application is limited due to the toxicity effect on mammalian cells^24^,^25^,^26^. Therefore, sequence modification of natural peptides with known activity is essential to enhance the antimicrobial activity and reduce the toxicity of peptides. In this study, KABT-AMP, a synthetic peptide and uperin 3.6, a natural peptide isolated from the Australian toadlet, *Uperoleia mjobergii* were selected as a template to design a new class of peptides^20^,^21^. Physicochemical parameters such as cationicity, hydrophobicity and amphipathicity were taken into consideration for peptide design (Table 1).

Hydrophobicity is one of the crucial parameters responsible for the antimicrobial activity of AMPs. It facilitates the AMP-membrane interaction and governs the extent of partition of AMPs into the cell membrane. Studies showed that enhancement of the overall hydrophobicity increased the antimicrobial potency of AMPs^27^,^28^,^29^. Based on GRAVY score, KU1 had the highest hydrophobicity (positive GRAVY score) among the hybrid peptides. However, the MIC values obtained show that KU2 was more potent than KU1 although KU2 is more hydrophilic (negative GRAVY score). By observing the helical projection of the peptides, we found that the enhancement of anticandidal activity of KU2 can be due to the number of tryptophan (Trp) residues incorporated in the peptide sequences. Tryptophan (Trp) is a hydrophobic residue that has a strong preference for the interfacial region of the lipid bilayers of the yeast cell membrane^30^. Replacement of Val^10^ at KU1 by Trp residue resulted in three Trp residues (Trp^3^, Trp^6^ and Trp^10^) positioned at the nonpolar face of KU2 and hence, enhanced the anchoring of the peptide to membrane (Figure 1b). By comparing KU2 and KU3, substitution of Asn^12^ by Lys residue at the hydrophilic face has no effect on the anticandidal activity (Figure 1c). KU4 demonstrated the least anticandidal potency among the hybrid peptides as a result of the substitution of hydrophobic Val^13^ by Lys residue which reduced the hydrophobicity of the peptide (Figure 1d). These results are consistent with a previous study^18^.

Cationic residues on the peptide promote the electrostatic attraction of the peptides to the anionic phospholipids in fungal membranes such as phosphatidylserine and phosphatidylinositol or wall components such as mannoproteins in yeast^31^,^32^. According to the MIC results of uperin 3.6 lysine-substituted analogues, substitution of a single lysine residue into the parent peptide (Upn-lys4) exhibited no improved activity against *C. albicans* strains and *C. krusei*. Upn-lys5 and Upn-lys6 displayed two-fold increase in potency against *C. albicans* as one or two Lys residues were substituted into the parent peptide. This suggested that further increase in the number of positive charge residue on Upn-lys6 is required as it may enhance the mediation of interaction of peptides with thick yeast cell walls.

Rapid killing effect is one of the important features of AMPs. Based on the results obtained (Figure 2), we suggest that the killing kinetics of the designed peptides correlated with their cationicity. KABT-AMP with a net charge of +10 demonstrated rapid killing rates with fungicidal effect (>99.99% killing) within 4 hours of incubation. KU4 (+7) reached the fungicidal endpoint at 6 hours of test period, showing better killing effect as compared to KU1 (+5), KU2 (+5) and KU3 (+6) although KU4 had the highest MIC value among the hybrid peptides (Figure 2a). In term of uperin 3.6 analogues, the killing effects were enhanced as the number of cationic lysine residues increased (Figure 2b). Previous studies on magainin analogues reported that increased cationicity increased the translocation of peptides across the membrane^33^. However, uperin 3.6 with +3 net charge displayed an exception in this case. It exhibited fungicidal effect after 4 hours of test period which is almost similar to the killing kinetic demonstrated by Upn-lys6. This is probably due to the presence of negatively charged aspartic acid, D in uperin 3.6, which may contribute to the electrostatic interaction of the peptide with yeast cell membrane.

*C. albicans* is the most common fungal species associated with biofilm formation^34^. Mature *Candida* biofilms display a complex three-dimensional structure consisting of a dense network of yeasts, hyphae and pseudohyphae and are frequently encountered on the surface of most implanted medical devices^34^. *Candida* biofilms are more resistant to host defense mechanism and antifungal agents such as fluconazole and amphotericin B. The resistance can be up to 1000-fold greater than planktonic cells, depending on both drug and yeast species^35^,^36^. For AMPs, Burrows *et al* demonstrated that the most potent peptide in their studies, dF 21-10K, was less effective at 5 × MIC and a concentration of 10 × MIC was required to eradicate biofilm cells completely^37^. In our study, although KU1, KU2 and KU3 showed potent anticandidal activity against planktonic cells, they were unable to reduce biofilm viability to 50% even at 4 × MIC (BECs > 4 × MIC) (Table 3). In contrast, KU4 and KABT-AMP with higher MIC values demonstrated potent antibiofilm activity. In term of uperin 3.6 analogues, we found that the BEC-2 values reduced as the number of lysine substituted increased. Hence, we suggest that the enhanced antibiofilm activity of the peptides against *C. albicans* may have a correlation with the increased cationicity of the peptides.

Drug combination therapy which involves the use of two or more drugs with separate mode of action can potentially improve the current available antimicrobial treatment. Combination regimens, in certain cases could potentially improve the efficacy, reduce toxic side effects and the cost of each individual drug and limit the development of antifungal resistance as shown in monotherapy^38^. A number of studies reported that AMPs exhibited synergistic effect with conventional antifungal agents^38^,^39^,^40^. In the current study, all peptide-amphotericin B combinations demonstrated synergistic activity (FICI ≤ 0.5) when tested on *C. albicans* SC5314 strain. For *C. albicans* ATCC 90028 strain, all peptides exhibited synergistic effects with amphotericin B with the exception of Upn-Lys6 (FICI 0.58) and uperin 3.6 (FICI 0.67). With regard to fluconazole, there were four peptide-fluconazole combinations (KU4, KABT-AMP, Upn-lys6 and uperin 3.6) and only one KU4-fluconazole combination displayed synergistic effects against *C. albicans* SC5314 strain and ATCC 90028 strain, respectively. In general, two compounds that interact in a synergistic way most probably exert antimicrobial activity in a different mechanism of action. Amphotericin B demonstrated fungicidal effect by binding to the fungal cell membrane ergosterol moiety and causing porosity on the fungal membrane. Fluconazole is a fungistatic azole-based antifungal which interferes with the key enzyme involved in the ergosterol biosynthesis and thereby inhibits the formation of ergosterol. In term of AMPs, various mechanisms of action have been proposed which include the ability of AMPs to damage cell membrane, interact with intracellular targets such as inhibition of cell wall, nucleic acid and protein synthesis, and induction of apoptosis^41^. Therefore, as the peptides synergistically interacted with fluconazole and amphotericin B, this might indicate that their mode of actions differ from both antifungals, and the synergistic effects could be attributed to the combination of two different mechanism of actions.

Although the peptides designed in this study demonstrated improved anticandidal activity, their therapeutic potential was hindered by toxicity. KU1, KU2 and KU3, which had the greatest hydrophobicity among hybrid peptides, demonstrated significant toxicity to red blood cells with low HC_10_ and HC_50_ values. Peptides with higher hydrophobicity tend to penetrate deeper into the hydrophobic core of red blood cell membrane and cause stronger haemolytic activities. In term of KU4, less haemolytic activity was observed due to the overall low hydrophobicity. Besides that, we found that nonpolar helix face of KU4 was disrupted by Lys^13^ residue and hence, reducing the penetration of the peptide into the cell membrane^27^,^42^. In term of uperin 3.6 analogues, no haemolytic activity was observed although they exhibited greater overall hydrophobicity compare to other hybrid peptides. Hence, the haemolytic activity was probably attributed to the tryptophan residues present in the hybrid peptides^18^. However, uperin 3.6 analogues induced significant toxicity against normal vaginal cell and esophagus epithelial cell lines although they did not lyse the erythrocytes. We propose that the mechanism of action of uperin 3.6 and its analogues could be organelle or nucleus dependent as they did not show toxicity effect on enucleated red blood cells^18^. Therefore, further investigations on these peptides are needed to elucidate the mechanisms of action of these peptides to kill *Candida* cells.

Molecular docking investigations enable clarifications of the binding mode of the peptides with the possible target proteins (sap1, sap5 and Exg). According to docking results, all peptides bound well with the target proteins since they demonstrated negative binding affinities to the target proteins (Supplementary data: Table S2). The interaction energy which is the sum of van Der Waals (VDW) and electrostatic interaction determines the interaction between peptides and target proteins. Based on the results of the interaction energy obtained, we suggest that KU3, KABT-AMP, Upn-lys5 and uperin 3.6 exhibit stronger interaction with sap5 as compared to sap1 and Exg.

## Conclusion

This study provides us a better understanding of the structure-activity relationship of antifungal peptides although the therapeutic applications of the peptides are limited due to toxicity. Several parameters such as hydrophobicity, the number of tryptophan residues, amphipathicity and cationicity are crucial for anticandidal activity of the peptides. We demonstrated that the antibiofilm activity and the killing kinetics are correlated with the cationicity of the peptides. The designed peptides also showed synergistic effects when used in combination with conventional antifungals and hence, this may result in reduced probability of resistance development in *Candida* spp. against the conventional antifungals. Further investigations are needed to elucidate the mechanism of action and mode of killing of the peptides.

## Methods

### Yeast strains and growth conditions

*C. albicans* strains SC5314 and ATCC 90028, *C. krusei* ATCC 6258 and *C. parapsilosis* ATCC 22019 were used in the study. The strains were stored at −80°C in nutrient broth supplemented with 2.0% glycerol stock. Prior to experimentation, each strain was streaked for single colonies on Sabouraud Dextrose (SDA) agar plates and incubated overnight at 37°C.

### Peptide synthesis

Peptides were synthesized to >90% purity by Mimotopes Pty Ltd (Clayton, Victoria, Australia. http://www.mimotopes.com/). The characterization and purity estimation of the peptides were performed by using electrospray mass spectrometry (ESMS) and reverse phase high performance liquid chromatography (RP-HPLC). All peptides were dissolved in sterilized water to 10 mg/ml and stored in aliquots at −20°C.

### MIC determinations

Antifungal activity of the designed peptides was determined according to a standardized broth microdilution method (Clinical and Laboratory Standards Institute (CLSI) document M27-A2). Briefly, the yeast colonies from 24- hour-old cultures of *Candida* species were picked and resuspended in 5 mL of sterile 0.145 mol/L saline and adjusted to cell density of 1 × 10^6^ to 5 × 10^6^ cells/ml. The yeast stock suspension was then diluted to obtain a starting inoculum of 5.0 × 10^2^ to 2.5 × 10^3^ cells/ml. Conventional antifungals such as amphotericin B (Sigma, USA) and fluconazole (Sigma, USA) were included in this study as controls and to compare the anticandidal activity with the designed peptides. The drugs were dissolved in DMSO and prepared to a stock concentration of 25 mg/L and 50 mg/L, respectively. Peptides and the antifungals were then serially diluted in RPMI 1640 growth medium buffered with morpholinepropanesulfonic acid (MOPS) in volume of 100 μL per well, giving final concentrations ranging from 256 mg/L to 0.5 mg/L in sterile U-bottomed 96-well polypropylene microplates. 100 μL of standardized yeast suspension was then added to each well. Plates were incubated for 48 hours at 37°C. The minimum inhibitory concentration (MIC) was defined as the lowest concentration that inhibited 90% growth of *Candida* species. The MICs were determined three times and each time in duplicate.

### Time killing assay

Killing kinetics of the peptides were determined by diluting the peptides with RPMI medium to a final concentration of 2 times the previously determined MIC for the strain to be tested. A fungal suspension was adjusted to a 0.5 McFarland turbidity standard with cell density of 1 × 10^6^ to 5 × 10^6^ cells/ml. 100 μL of adjusted yeast suspension was exposed to 900 μL of RPMI medium containing peptide, yielding a starting inoculum of approximately 10^5^ cells/ml. The solutions were incubated at 37°C with agitation. At the predetermined time interval of 0, 1, 2, 4 and 6 hours, 10 μL or 30 μL of the samples were removed, serially diluted with phosphate-buffered saline (PBS) and plated on SDA plates to allow colony counts after 24 hours of incubation. Controls for yeast growth and antifungal agents were also performed. The lower limit of accurate and reproducible quantitation was 100 CFU/ml. Results were obtained from three independent experiments.

### Biofilm reduction assay

Biofilms of *C. albicans* were formed on commercially available pre-sterilized, polystyrene, flat bottomed 96 well microtitre plates as described by Jin *et al* with modification^43^. Firstly, a standardized yeast suspension (10^7^ cells/ml) of *C. albicans* SC5314 was prepared by suspending colonies from 24-hours old culture in RPMI 1640 medium and adjusted to an optical density of 0.38–0.39 at 520 nm. 100 μL of yeast suspension was dispensed into each well of a microtitre plate using a multichannel pipette and the plates were incubated in a shaking incubator at 37°C with 75 rpm for 90 minutes to allow adherence of yeast on the surface of each well. After the adhesion phase, the non-adherent cells were removed and each well was washed twice with 150 μL PBS. 100 μL of RPMI 1640 medium was transferred to each washed well and the plates were incubated at 37°C in a shaking incubator at 75 rpm for 24 hours to allow biofilm formation. Following biofilm phase, the medium was aspirated and each well was washed twice gently with 200 μL PBS to remove non-adherent cells. Residual PBS was removed by inverting the plates over an absorbent paper before addition of peptides. 200 μL of each peptide with concentrations ranging from 1 to 4 times of the MIC determined previously was added to respective wells and the plates were incubated as described above for 24 h. Antifungal agent-free wells and biofilm-free wells were included as positive and negative controls. After treatment with peptides, the medium was removed and each well was washed twice with 200 μL PBS. The biofilm formation was quantified by using XTT reduction assay as described below. Each experiment was repeated three times in duplicate.

XTT reduction assay was performed according to the method adapted from Jin *et al*.^44^ Briefly, XTT (Sigma) was dissolved to a concentration of 1 mg/ml using sterile PBS. XTT solution was then filter-sterilized using a 0.22 μm-pore-size filter and kept at −70°C prior to use. 0.4 mM menadione solution was prepared by dissolving menadione (Sigma) with acetone. The menadione solution was filter-sterilized and kept at −70°C before use. Prior to each assay, XTT solution was thawed and mixed with menadione solution at a volume ratio of 20:1. After washing, a total volume of 200 μL of XTT-menadione mixture (158 μL of PBS, 40 μL of XTT and 2 μL of menadione) was added to each prewashed biofilm and control wells. The plates were incubated in the dark for 2 hours at 37°C. 100 μL of the solution was transferred to new wells and the colorimetric change in the solution was measured using a microtiter plate reader at 490 nm. Antibiofilm activity of the peptides and conventional antifungals was expressed as the biofilm-eradicating concentration 2 (BEC-2), which is defined as the minimum concentration of the peptides resulting in 50% reduction of the biofilm viability compared to the growth control.

### Chequerboard antifungal analysis

Peptide-peptide and peptide-antibiotic interactions were assessed in sterile U-bottomed 96-well polystyrene microplates using a chequerboard method with minor modification. 50 μL of eight serial twofold dilutions of peptide and ten serial twofold dilutions of antifungal agent with beginning concentration at 4 × MIC were prepared in each well. A fixed concentration of 0.25 × MIC of the peptide was added in a volume of 50 μL into each column. Each well of the plates was inoculated with 5.0 × 10^2^ to 2.5 × 10^3^ cells/ml of the yeast suspension. The plates were incubated at 37°C for 48 hours. The fractional inhibitory concentration (FIC), defined as MIC of the drug used in combination divided by the MIC of drug tested alone, was determined^45^. The effects of the peptide-peptide and peptide-antibiotic combinations were expressed in term of FIC index (FICI), where by
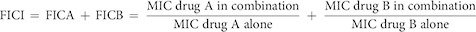


The FICI ≤ 0.5 was interpreted as synergism; while FICI > 0.5–4 was an indication of no interaction; and FICI > 4 was interpreted as antagonism.

### Haemolytic assay

Freshly collected human blood with heparin was centrifuged to remove the buffy coat. The erythrocytes were washed three times with PBS, centrifuged for 10 min at 2000 rpm at 10°C, and resuspended in PBS to 4% (vol/vol). Peptides were serially diluted in PBS to a concentration ranging from 256 mg/L to 0.5 mg/L (100 μL) in U-bottomed 96-well polystyrene microplates and 100 μL of the erythrocyte suspension was added to each well. PBS and 0.1% Triton X-100 were used as agents to induce 0 and 100% haemolysis, respectively. Plates were incubated for 1 hour at 37°C and centrifuged at 2000 rpm for 10 min. The supernatant (100 μL) was transferred to a 96-well flat-bottomed polystyrene plate, and the release of haemoglobin was monitored by measuring the absorbance at 560 nm using a microplate reader. Results were repeated three times and each time in duplicate. The percentage of haemolysis was calculated using the following formula:
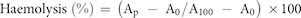
where A_p_ and A_100_ represent the absorbance of erythrocytes treated with peptide and 0.1% Triton X-100. A_0_ is the absorbance of erythrocytes without treatment.

### Cytotoxicity against normal human cell lines

The human normal vaginal epithelial cell line VK2/E6E7 (ATCC® CRL-2616™) and human normal esophagus epithelial cell line Het-1A (ATCC® CRL-2692™) were used for cytotoxicity testing. VK2/E6E7 cell line was grown in Keratinocyte-Serum Free medium (GIBCO-BRL 17005-042) supplemented with 0.1 ng/ml human recombinant EGF, 0.05 mg/ml bovine pituitary extract, and 44.1 mg/L calcium chloride. Het-1A cell line was grown in flask coated with 0.01 mg/ml fibronectin, 0.03 mg/ml bovine collagen type 1 and 0.01 mg/ml bovine serum albumin and maintained in BEBM medium supplemented with BEGM (SingleQuots) except gentamicin-amphotericin B mix. VK2/E6E7 and Het-1A cells were seeded overnight at 1 × 10^4^ cells/well in 96-well flat bottom microplates and treated with 100 μL of serial diluted peptides at concentrations ranging from 256 mg/L to 0.5 mg/L. Plates were incubated for 24, 48 and 72 hours at 37°C under 5% CO_2_. Cell viability was measured by using CellTiter 96® Aqueous Non-Radioactive Cell Proliferation Assay (Promega, USA) and the colourimetric changes were determined at 490 nm using a microplate reader. IC_50_ was defined as the peptide concentration required for 50% reduction in cell viability. Results were recorded by averaging three repeated experiments and each experiment was performed in duplicate.

## Molecular docking study

The Protein Data Bank (PDB) files of Sap1 (2QZW), Sap5 (2QZX) and exo-beta-(1, 3)-glucanase (1CZ1) with respective resolution of 2.05 Å, 2.50 Å, and 1.85 Å were obtained from the RSCB protein data bank. Tertiary structure of the designed and template peptides was predicted using peptide tertiary structure prediction server (Pepstr, http://www.imtech.res.in/raghava/pepstr/)^46^. All selected target proteins and peptides underwent minimization by applying the CHARMm forcefield with Memony Rone partial charge using Discovery Studio Client 2.5, San Diego: Accelrys Software Inc^47^. The docking procedure was conducted by Autodock Vina with rigid docking^48^. Complex structures with strong binding affinity were further minimized and analyzed. The interaction energy of the complex within 3 Ǻ was explored by interaction energy protocol of Discovery Studio Client 2.5, San Diego: Accelrys Software Inc.

## Supplementary Material

Supplementary Information Supplementary Information

## Figures and Tables

**Table 1:**
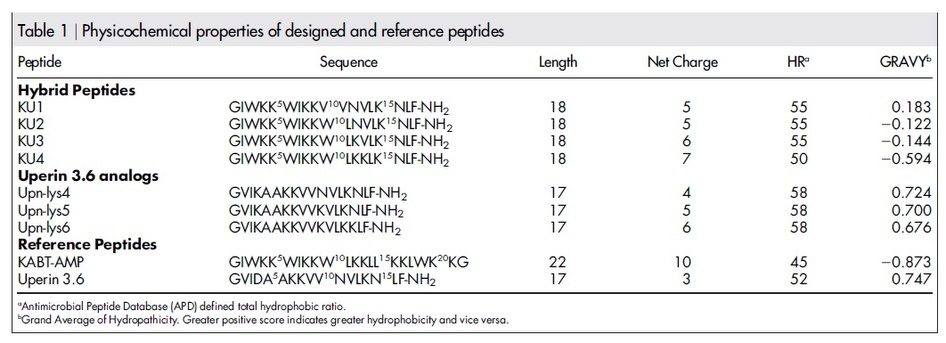
Physicochemical properties of designed and reference peptides.

**Table 2:** Antimicrobial activities (MIC) of peptides and conventional antifungals against *Candida* strains

	Minimum Inhibitory Concentration (MIC)(mg/L)			
	*Candida albicans*		*Candida krusei*	*Candida parapsilosis*
Peptide or antifungal	SC 5314	ATCC 90028	ATCC 6258	ATCC 22019
KU1	16	32	32	16
KU2	8	16	16	8
KU3	8	16	16	8
KU4	32	64	16	32
Upn-lys4	64	128	64	64
Upn-lys5	32	64	32	32
Upn-lys6	32	64	32	16
KABT-AMP	32	64	64	64
Uperin 3.6	64	128	64	128
Fluconazole	1	2	64	1
Amphotericin B	1	0.5	1	1

**Table 3:** Antibiofilm activity of designed peptides and conventional antifungals against *Candida albicans*

Peptides	BEC-2 values (mg/L)
KU1	>64
KU2	>32
KU3	>32
KU4	96
Upn-lys4	192
Upn-lys5	128
Upn-lys6	96
Uperin 3.6	192
KABT-AMP	64
Fluconazole	>4
Amphotericin B	<1

**Table 4:** Haemolytic activity of peptides and conventional antifungals on human erythrocytes

Peptide or antifungal	Haemolytic activity (mg/L)	
	HC_10_ a	HC_50_ a
KU1	8.07 ± 1.60	58.19 ± 2.04
KU2	7.33 ± 0.73	57.71 ± 9.80
KU3	5.65 ± 0.85	55.20 ± 11.90
KU4	49.38 ± 5.14	>256
Upn-lys4	>256	>256
Upn-lys5	>256	>256
Upn-lys6	>256	>256
KABT-AMP	4.67 ± 2.09	81.23 ± 8.92
Uperin 3.6	>256	>256
Fluconazole	>256	>256
Amphotericin B	5.79 ± 1.59	60.94 ± 15.88

aHC_10_ and HC _50_ are concentration that causes 10% and 50% haemolysis of human erythrocytes measured after 1 hour of incubation of erythrocytes with peptides.

**Table 5:** Cytotoxicity of peptides and conventional antifungals on VK2/E6E7 and Het-1A cell lines

Peptide or antifungal	IC_50_ (mg/L)					
	VK2/E6E7 cell line			Het-1A cell line		
	24 hrs	48 hrs	72 hrs	24 hrs	48 hrs	72 hrs
**KU1**	4.28 ± 0.60	4.71 ± 0.22	7.84 ± 1.99	3.61 ± 0.19	4.59 ± 0.26	7.27 ± 1.17
**KU2**	5.42 ± 0.49	7.01 ± 0.84	11.38 ± 1.75	5.51 ± 0.44	6.03 ± 0.43	8.89 ± 1.40
**KU3**	4.43 ± 0.39	5.32 ± 0.84	7.83 ± 1.41	4.33 ± 0.52	4.62 ± 0.65	6.36 ± 1.34
**KU4**	8.75 ± 1.04	10.55 ± 2.33	19.46 ± 4.29	9.75 ± 1.34	10.47 ± 0.50	14.53 ± 1.58
**Upn-lys4**	15.45 ± 2.64	16.55 ± 0.55	19.98 ± 1.84	14.12 ± 4.35	14.71 ± 4.44	23.64 ± 2.82
**Upn-lys5**	8.67 ± 2.21	10.28 ± 4.53	9.12 ± 1.20	9.73 ± 1.19	9.83 ± 1.40	15.73 ± 0.39
**Upn-lys6**	9.07 ± 1.92	10.04 ± 3.27	9.87 ± 1.46	7.49 ± 1.77	7.92 ± 1.22	11.20 ± 0.86
**KABT-AMP**	6.03 ± 1.86	5.86 ± 1.51	7.14 ± 1.80	3.01 ± 0.59	3.31 ± 0.61	4.45 ± 0.71
**Uperin 3.6**	17.14 ± 4.32	23.59 ± 1.18	24.69 ± 6.02	17.60 ± 2.52	17.94 ± 1.96	23.23 ± 2.98
**Fluconazole**	>256	>256	>256	>256	>256	>256
**Amphotericin B**	8.13 ± 4.54	7.64 ± 2.95	15.31 ± 3.72	9.91 ± 3.35	7.78 ± 1.70	9.10 ± 0.98
