# Supplementary material for: Activity of Novel Synthetic Peptides against Candida albicans
Source: Sci Rep. 2015 May 12;5:9657. doi: 10.1038/srep09657 (PMC4603303; doi:10.1038/srep09657)
Supplement: Supplementary Information [file srep09657-s1.docx]

**Activity of Novel Synthetic Peptides against *Candida albicans***

Kah Yean Lum^1^, Sun Tee Tay^1^, Cheng Foh Le^2^, Vannajan Sanghiran Lee^3^, Nadia Hanim Sabri^3^ , Rukumani Devi Velayuthan^1^, Hamimah Hassan^1^ and Shamala Devi Sekaran^1*^

^1^Department of Medical Microbiology, Faculty of Medicine, University of Malaya, Kuala Lumpur, Malaysia.

^2^School of Pharmacy, Faculty of Science, University Nottingham Malaysia Campus, Semenyih, Selangor, Malaysia

^3^Department of Chemistry, Faculty of Science, University of Malaya, Kuala Lumpur.

Corresponding author. Department of Medical Microbiology, Faculty of Medicine, University of Malaya, Kuala Lumpur, Malaysia. Phone: +603-7967 5759. Fax: +603-7967 6672. E-mail: [shamala@um.edu.my](mailto:shamala@um.edu.my)

**Table S1:** Growth inhibitory effect of peptide-peptide and peptide-antifungal combinations against two *C.albicans* strains.

| Combination | | SC 5314 | | | ATCC 90028 | | |
| --- | --- | --- | --- | --- | --- | --- | --- |
| A | **B** | MIC_A_ | MIC_B_ | FIC index | MIC_A_ | MIC_B_ | FIC index |
| KU1 | KU2 | 4 | 8 | 1.25 | 8 | 16 | 1.25 |
|  | KU3 | 4 | 8 | 1.25 | 8 | 16 | 1.25 |
|  | KU4 | 4 | 32 | 1.25 | 8 | 53.33 | 1.08 |
|  | Upn-lys4 | 4 | 128 | 2.25 | 8 | 128 | 1.25 |
|  | Upn-lys5 | 4 | 64 | 2.25 | 8 | 64 | 1.25 |
|  | Upn-lys6 | 4 | 32 | 1.25 | 8 | 37.33 | 0.83 |
|  | KABT-AMP | 4 | 32 | 1.25 | 8 | 64 | 1.25 |
|  | Uperin 3.6 | 4 | 128 | 2.25 | 8 | 1.33 | 0.92 |
|  | Fluconazole | 4 | 1 | 1.25 | 8 | 96 | 1 |
|  | Amphotericin B | **4** | **0.06** | **0.31** | **8** | **0.125** | **0.50** |
|  |  |  |  |  |  |  |  |
| KU2 | KU3 | 2 | 7.33 | 1.17 | 4 | 16 | 1.25 |
|  | KU4 | 2 | 32 | 1.25 | 4 | 32 | 1.25 |
|  | Upn-lys4 | 2 | 128 | 2.25 | 4 | 128 | 1.25 |
|  | Upn-lys5 | 2 | 64 | 2.25 | 4 | 64 | 1.25 |
|  | Upn-lys6 | 2 | 32 | 1.25 | 4 | 52 | 1.06 |
|  | KABT-AMP | 2 | 42.67 | 1.58 | 4 | 64 | 1.25 |
|  | Uperin 3.6 | 2 | 85.33 | 1.58 | 4 | 128 | 1.25 |
|  | Fluconazole | 2 | 0.67 | 0.92 | 4 | 1.33 | 0.92 |
|  | Amphotericin B | **2** | **0.06** | **0.31** | **4** | **0.125** | **0.50** |
|  |  |  |  |  |  |  |  |
| KU3 | KU4 | 2 | 32 | 1.25 | 4 | 48 | 1 |
|  | Upn-lys4 | 2 | 128 | 2.25 | 4 | 128 | 1.25 |
|  | Upn-lys5 | 2 | 64 | 2.25 | 4 | 64 | 1.25 |
|  | Upn-lys6 | 2 | 32 | 1.25 | 4 | 64 | 1.25 |
|  | KABT-AMP | 2 | 32 | 1.25 | 4 | 64 | 1.25 |
|  | Uperin 3.6 | 2 | 74.67 | 1.42 | 4 | 128 | 1.25 |
|  | Fluconazole | 2 | 0.67 | 0.92 | 4 | 1 | 0.75 |
|  | Amphotericin B | **2** | **0.06** | **0.31** | **4** | **0.125** | **0.50** |
|  |  |  |  |  |  |  |  |
| KU4 | Upn-lys4 | 8 | 128 | 2.25 | 16 | 128 | 1.25 |
|  | Upn-lys5 | 8 | 64 | 2.25 | 16 | 64 | 1.25 |
|  | Upn-lys6 | 8 | 32 | 1.25 | 16 | 64 | 1.25 |
|  | KABT-AMP | 8 | 32 | 1.25 | 16 | 32 | 0.75 |
|  | Uperin 3.6 | 8 | 149.33 | 2.58 | 16 | 128 | 1.25 |
|  | Fluconazole | **8** | **0.13** | **0.38** | **16** | **0.19** | **0.34** |
|  | Amphotericin B | **8** | **0.06** | **0.31** | **16** | **0.125** | **0.50** |
|  |  |  |  |  |  |  |  |
| Upn-lys4 | Upn-lys5 | 16 | 42.67 | 1.58 | 32 | 58.67 | 1.17 |
|  | Upn-lys6 | 16 | 16 | 0.75 | 32 | 32 | 0.75 |
|  | KABT-AMP | 16 | 32 | 1.25 | 32 | 48 | 1 |
|  | Uperin 3.6 | 16 | 42.67 | 0.92 | 32 | 64 | 0.75 |
|  | Fluconazole | 16 | 0.33 | 0.58 | 32 | 1.67 | 1.08 |
|  | Amphotericin B | **16** | **0.10** | **0.35** | **32** | **0.125** | **0.50** |
|  |  |  |  |  |  |  |  |
| Upn-lys5 | Upn-lys6 | 8 | 16 | 0.75 | 16 | 32 | 0.75 |
|  | KABT-AMP | 8 | 37.33 | 1.42 | 16 | 37.33 | 0.83 |
|  | Uperin 3.6 | 8 | 64 | 1.25 | 16 | 64 | 0.75 |
|  | Fluconazole | 8 | 0.5 | 0.75 | 16 | 2 | 1.25 |
|  | Amphotericin B | **8** | **0.05** | **0.30** | **16** | **0.125** | **0.50** |
|  |  |  |  |  |  |  |  |
| Upn-lys6 | KABT-AMP | 8 | 32 | 1.25 | 16 | 32 | 0.75 |
|  | Uperin 3.6 | 8 | 85.33 | 1.58 | 16 | 64 | 0.75 |
|  | Fluconazole | 8 | **0.17** | **0.42** | 16 | 2 | 1.25 |
|  | Amphotericin B | **8** | **0.05** | **0.30** | 16 | 0.17 | 0.58 |
|  |  |  |  |  |  |  |  |
| KABT-AMP | Uperin 3.6 | 8 | 74.67 | 1.42 | 16 | 128 | 1.25 |
|  | Fluconazole | 8 | **0.25** | **0.50** | 16 | 0.83 | 0.67 |
|  | Amphotericin B | **8** | **0.04** | **0.29** | **16** | **0.10** | **0.46** |
|  |  |  |  |  |  |  |  |
| Uperin 3.6 | Fluconazole | **16** | **0.25** | **0.5** | 32 | 0.67 | 0.58 |
|  | Amphotericin B | **16** | **0.08** | **0.33** | 32 | 0.21 | 0.67 |

**Table S2:** Binding affinity of peptides with target proteins by autodock Vina.

| Peptide | Binding affinity (kcal/mol) | | |
| --- | --- | --- | --- |
|  | **2QZW*** | **2QZX*** | **1CZ1*** |
| KU1 | -11.1-(-8.3) | -10.1-(-7.4) | -11.8-(9.0) |
| KU2 | -12.2-(-11.0) | -9.2-(-7.0) | -11.9-(-9.0) |
| KU3 | -12.6- (-11.2) | -9.6-(-7.0) | -12.8-(-10.0) |
| KU4 | -11.1-(-8.2) | -11.9-(-9.2) | -10.7-(-8.6) |
| KABT-AMP | -9.5- (-7.0) | -9.5-(-7.0) | -11.0-(-8.3) |
| Upn-lys4 | -11.2-(-8.9) | -12.6-(-10.1) | -11.5-(-9.2) |
| Upn-lys5 | -10.5-(-8.8) | -12.3-(-9.3) | 11.6-(-8.9) |
| Upn-lys6 | -10.4-(-8.1) | -12.1-(-9.2) | -11.6-(-8.8) |
| Uperin 3.6 | -10.5-(-8.7) | -11.7-(-9.4) | -10.8-(-9.3) |

* 2QZW, 2QZX and 1CZ1 represent the PDB ID of sap1, sap5 and exo-β-(1,3)-glucanases, respectively.

| Residue  **TABLE S3**: Contribution of the interactions energy in kcal/mol of the sap1 (PDB ID: 2QZW) in the binding residues within 3Å as highlighted in bold letter. | Interaction Energy (IE) | VDW | Electrostatic | | Residue | Interaction Energy (IE) | VDW | Electrostatic | | Residue | Interaction Energy (IE) | VDW | Electrostatic | | Residue | Interaction Energy (IE) | | VDW | Electrostatic | |
| --- | --- | --- | --- | --- | --- | --- | --- | --- | --- | --- | --- | --- | --- | --- | --- | --- | --- | --- | --- | --- |
| KABT-AMP GIWKKWIKKWLKKLLKKLWKKG-NH2 | | | | | KU3 GIWKKWIKKWLKVLKNLF-NH2 | | | | | Uperin 3.6  GVIDAAKKVVNVLKNLF-NH2 | | | | | Upn-Lys5  GVIKAAKKVVKVLKNLF-NH2 | | | | | |
| **A_SER36** | **1.488** | **-0.264** | | **1.753** | A_SER36 | -9.115 | -0.314 | | -8.801 | A_ILE82 | -1.716 | -0.001 | -1.715 | | **A_ILE82** | | **0.591** | **-1.440** | | **2.031** |
| **A_ASP37** | **-98.686** | **-0.278** | | **-98.408** | A_ASP37 | -75.095 | -0.456 | | -74.639 | A_GLY83 | -2.446 | -0.001 | -2.444 | | **A_GLY83** | | **-4.604** | **0.353** | | **-4.957** |
| **A_TYR81** | **-10.099** | **-2.325** | | **-7.773** | **A_TYR81** | **-1.635** | **-0.076** | | **-1.559** | A_TYR84 | -3.042 | -0.168 | -2.874 | | **A_TYR84** | | **-11.850** | **-4.545** | | **-7.305** |
| **A_ILE82** | **-4.173** | **-2.446** | | **-1.727** | **A_ILE82** | **5.598** | **-1.544** | | **7.142** | A_GLY85 | -11.090 | -0.396 | -10.694 | | **A_GLY85** | | **-11.341** | **-1.648** | | **-9.693** |
| **A_GLY83** | **-15.092** | **-3.796** | | **-11.297** | **A_GLY83** | **-10.997** | **-0.639** | | **-10.358** | **A_ASP86** | **-37.943** | **-3.641** | **-34.303** | | **A_ASP86** | | **-109.574** | **-2.519** | | **-107.055** |
| **A_TYR84** | **-15.323** | **-2.552** | | **-12.771** | A_TYR84 | -6.427 | -0.807 | | -5.620 | A_ASN131 | 0.094 | 0.000 | 0.094 | | **A_ASN131** | | **-24.878** | **-3.277** | | **-21.600** |
| **A_GLY85** | **-6.872** | **-1.375** | | **-5.497** | A_GLY85 | -5.875 | -0.415 | | -5.461 | A_GLU132 | 0.000 | 0.000 | 0.000 | | **A_GLU132** | | **-27.977** | **-1.011** | | **-26.966** |
| **A_SER88** | **-36.689** | **-0.425** | | **-36.264** | A_SER88 | -17.570 | -0.023 | | -17.547 | A_ALA133 | 0.000 | 0.000 | 0.000 | | **A_ALA133** | | **-3.324** | **-0.946** | | **-2.378** |
| **A_ASN131** | **-35.330** | **-0.968** | | **-34.362** | **A_ASN131** | **-26.058** | **-2.878** | | **-23.180** | A_ASP191 | 0.000 | 0.000 | 0.000 | | **A_ASP191** | | **-146.529** | **3.373** | | **-149.902** |
| A_GLU132 | -4.848 | -0.546 | | -4.302 | **A_GLU132** | **-16.197** | **-1.580** | | **-14.617** | A_ARG192 | 0.502 | 0.000 | 0.502 | | **A_ARG192** | | **84.814** | **-6.899** | | **91.713** |
| **A_ALA133** | **2.446** | **-1.445** | | **3.891** | **A_ALA133** | **-0.140** | **-1.517** | | **1.376** | **A_THR222** | **-15.210** | **-2.825** | **-12.384** | | A_THR222 | | -5.038 | -0.092 | | -4.946 |
| **A_ASP191** | **-123.316** | **-0.106** | | **-123.210** | A_ASP191 | -32.439 | -0.200 | | -32.239 | **A_ILE223** | **-9.462** | **-2.856** | **-6.605** | | **A_ILE223** | | **-4.939** | **-1.195** | | **-3.744** |
| **A_ARG192** | **120.796** | **-4.057** | | **124.853** | **A_ARG192** | **81.026** | **-5.213** | | **86.239** | **A_TYR225** | **-14.089** | **-3.772** | **-10.317** | | **A_TYR225** | | **-9.758** | **-2.267** | | **-7.491** |
| **A_GLU193** | **-163.836** | **-0.702** | | **-163.134** | **A_GLU193** | **-143.609** | **-1.371** | | **-142.238** | **A_GLN228** | **-36.265** | **-1.289** | **-34.975** | | A_GLN228 | | -7.896 | -0.126 | | -7.770 |
| **A_ARG195** | **124.867** | **-1.932** | | **126.799** | A_ARG195 | 89.456 | -0.805 | | 90.261 | **A_ASP245** | **-56.872** | **-1.332** | **-55.540** | | A_ASP245 | | -28.848 | -0.339 | | -28.509 |
| **A_ASN212** | **-13.865** | **-0.771** | | **-13.094** | A_ASN212 | -11.450 | -0.060 | | -11.390 | **A_HIS249** | **24.208** | **-5.410** | **29.617** | | **A_HIS249** | | **14.315** | **-4.251** | | **18.567** |
| **A_ASP214** | **-90.970** | **1.409** | | **-92.379** | A_ASP214 | -46.602 | -0.163 | | -46.439 | **A_THR250** | **-21.342** | **-2.408** | **-18.934** | | A_THR250 | | -11.505 | -1.501 | | -10.003 |
| **A_GLN227** | **-0.041** | **-2.174** | | **2.132** | **A_GLN227** | **-27.213** | **-3.112** | | **-24.101** | **A_GLN295** | **-2.522** | **-0.578** | **-1.944** | | A_GLN295 | | -4.836 | -0.458 | | -4.378 |
| A_GLN228 | 4.004 | -0.194 | | 4.198 | **A_GLN228** | **-6.528** | **-1.981** | | **-4.547** | **A_LEU297** | **-10.813** | **-0.613** | **-10.199** | | A_LEU297 | | 1.696 | -0.126 | | 1.822 |
| **A_ASP229** | **-108.994** | **1.743** | | **-110.737** | **A_ASP229** | **-152.364** | **-0.729** | | **-151.635** | A_GLY299 | -9.642 | -1.005 | -8.637 | | **A_GLY299** | | **-6.226** | **-1.253** | | **-4.973** |
| A_ILE300 | -1.557 | -0.034 | | -1.523 | **A_ILE300** | **-2.348** | **-1.058** | | **-1.290** | **A_ILE300** | **-19.121** | **-5.650** | **-13.471** | | A_ILE300 | | -10.609 | -1.633 | | -8.977 |
| **A_SER301** | **-9.711** | **-0.919** | | **-8.792** | A_SER301 | -3.336 | -0.678 | | -2.658 | **A_SER301** | **-17.143** | **-3.588** | **-13.555** | | **A_SER301** | | **-25.709** | **-1.722** | | **-23.988** |
| **A_ASP302** | **-170.607** | **-4.204** | | **-166.403** | **A_ASP302** | **-203.582** | **-7.052** | | **-196.530** | **A_ASP302** | **-80.277** | **-2.113** | **-78.164** | | A_ASP302 | | -87.720 | -2.284 | | -85.437 |
| A_ALA303 | -15.924 | -2.416 | | -13.508 | **A_ALA303** | **-9.039** | **-3.246** | | **-5.793** | A_ALA303 | -2.844 | -0.225 | -2.618 | | **A_ALA303** | | **-8.075** | **-1.839** | | **-6.235** |
| A_ASN304 | -7.627 | -0.635 | | -6.992 | **A_ASN304** | **8.557** | **-1.093** | | **9.650** | A_ILE305 | -0.880 | -0.113 | -0.767 | | **A_ILE305** | | **-3.782** | **-0.733** | | **-3.049** |
| Total within 3(A) | -506.096 | -29.301 | | -476.795 | Total within 3(A) | -504.530 | -33.089 | | -471.442 | Total within 3(A) | -296.850 | -36.075 | | -260.775 | Total within 3(A) | | -298.846 | -31.820 | | -267.026 |
| Total (IE) | -679.960 | -31.412 | | -648.549 | Total (IE) | -622.983 | -37.009 | | -585.974 | Total (IE) | -327.915 | -37.985 | | -289.929 | Total (IE) | | -453.603 | -38.379 | | -415.224 |

| Residue  **TABLE S4**: Contribution of the interactions energy in kcal/mol of the sap5 (PDB: 2QZX) in the binding residues within 3Å as highlighted in bold letter. | Interaction Energy (IE) | VDW | Electrostatic | | Residue | Interaction Energy (IE) | VDW | Electrostatic | | Residue | Interaction Energy (IE) | | VDW | Electrostatic | | Residue | | Interaction Energy (IE) | VDW | | Electrostatic | |
| --- | --- | --- | --- | --- | --- | --- | --- | --- | --- | --- | --- | --- | --- | --- | --- | --- | --- | --- | --- | --- | --- | --- |
| KABT-AMP GIWKKWIKKWLKKLLKKLWKKG-NH2 | | | | | KU3 GIWKKWIKKWLKVLKNLF-NH2 | | | | | Uperin 3.6  GVIDAAKKVVNVLKNLF-NH2 | | | | | | Upn-Lys5  GVIKAAKKVVKVLKNLF-NH2 | | | | | | |
| **A_ASN9** | **-16.888** | **-0.009** | | **-16.879** | **A_ASN9** | **-16.481** | **-1.014** | | **-15.468** | A_GLU10 | | -45.973 | -0.201 | -45.772 | | **A_GLU10** | | **-119.582** | **-0.214** | | | **-119.368** |
| **A_GLU10** | **-18.915** | **-0.028** | | **-18.887** | A_GLU10 | -85.577 | -0.397 | | -85.180 | A_ALA11 | | -11.772 | -0.169 | -11.602 | | **A_ALA11** | | **-5.495** | **-1.239** | | | **-4.256** |
| **A_ALA11** | **-2.737** | **-0.041** | | **-2.696** | **A_ALA11** | **-18.261** | **-2.154** | | **-16.107** | **A_ILE12** | | **-15.484** | **-5.532** | **-9.952** | | **A_ILE12** | | **-11.070** | **-6.032** | | | **-5.039** |
| **A_ILE12** | **-4.821** | **-3.690** | | **-1.131** | **A_ILE12** | **-28.238** | **-7.537** | | **-20.701** | **A_THR13** | | **-1.312** | **-3.311** | **2.000** | | **A_THR13** | | **-5.582** | **-4.531** | | | **-1.051** |
| **A_THR13** | **-7.188** | **-1.253** | | **-5.936** | **A_THR13** | **-16.882** | **-4.084** | | **-12.797** | **A_ILE30** | | **-13.619** | **-3.773** | **-9.846** | | A_ILE30 | | 3.537 | -1.790 | | | 5.328 |
| A_TYR14 | -1.637 | -0.162 | | -1.475 | **A_TYR14** | **-42.756** | **-2.113** | | **-40.643** | **A_VAL31** | | **-10.406** | **1.322** | **-11.728** | | A_VAL31 | | -10.240 | -0.084 | | | -10.156 |
| **A_ILE30** | **-12.617** | **-2.919** | | **-9.698** | **A_ILE30** | **-19.065** | **-0.456** | | **-18.609** | **A_ASP32** | | **-140.932** | **-2.353** | **-138.579** | | **A_ASP32** | | **-166.848** | **2.994** | | | **-169.842** |
| **A_VAL31** | **-18.307** | **-0.695** | | **-17.612** | **A_VAL31** | **-12.368** | **-1.228** | | **-11.141** | **A_GLY34** | | **-20.943** | **1.554** | **-22.497** | | **A_GLY34** | | **8.739** | **-2.403** | | | **11.143** |
| **A_ASP32** | **-169.219** | **2.437** | | **-171.656** | **A_ASP32** | **-120.992** | **1.791** | | **-122.783** | **A_SER35** | | **-4.505** | **-6.318** | **1.813** | | **A_SER35** | | **-4.068** | **-3.461** | | | **-0.607** |
| **A_GLY34** | **-1.464** | **1.123** | | **-2.587** | A_GLY34 | -23.230 | -0.476 | | -22.754 | **A_SER36** | | **-7.291** | **-2.526** | **-4.765** | | A_SER36 | | -9.174 | -0.588 | | | -8.586 |
| **A_SER35** | **-14.518** | **-5.800** | | **-8.718** | **A_SER35** | **-10.333** | **-4.531** | | **-5.802** | **A_ASP37** | | **-76.583** | **-1.880** | **-74.703** | | A_ASP37 | | -113.491 | -0.344 | | | -113.147 |
| A_SER36 | -15.691 | -1.864 | | -13.827 | **A_SER36** | **-26.215** | **-1.539** | | **-24.676** | **A_TRP39** | | **-13.851** | **-1.303** | **-12.548** | | **A_TRP39** | | **2.411** | **-0.894** | | | **3.306** |
| **A_ASP37** | **-144.050** | **0.067** | | **-144.117** | **A_ASP37** | **-177.009** | **1.299** | | **-178.308** | **A_LYS50** | | **-5.901** | **-1.856** | **-4.045** | | **A_LYS50** | | **53.051** | **-1.616** | | | **54.667** |
| A_TRP39 | -14.812 | -0.912 | | -13.900 | **A_TRP39** | **1.523** | **-1.150** | | **2.673** | **A_TRP51** | | **-19.188** | **-8.129** | **-11.059** | | **A_TRP51** | | **-10.637** | **-5.425** | | | **-5.212** |
| **A_LYS50** | **79.448** | **-2.961** | | **82.409** | A_LYS50 | 76.146 | -0.965 | | 77.111 | **A_ARG52** | | **-19.933** | **-4.281** | **-15.651** | | A_ARG52 | | -19.999 | -0.142 | | | -19.857 |
| **A_TRP51** | **-17.354** | **-4.581** | | **-12.773** | **A_TRP51** | **-5.160** | **-3.445** | | **-1.714** | **A_GLY53** | | **-4.225** | **-0.925** | **-3.300** | | A_GLY53 | | -10.914 | -0.070 | | | -10.845 |
| **A_ASP54** | **-119.378** | **-0.077** | | **-119.301** | **A_ASP54** | **-137.798** | **2.823** | | **-140.621** | **A_ILE82** | | **-10.172** | **-2.085** | **-8.086** | | A_ILE82 | | -8.428 | -0.943 | | | -7.485 |
| **A_PHE80** | **-4.849** | **-0.968** | | **-3.881** | **A_PHE80** | **1.944** | **-0.601** | | **2.545** | **A_LYS83** | | **21.230** | **-4.125** | **25.356** | | **A_LYS83** | | **24.392** | **-3.870** | | | **28.262** |
| **A_ILE82** | **5.391** | **-1.453** | | **6.845** | **A_ILE82** | **-10.050** | **-4.562** | | **-5.489** | **A_TYR84** | | **-6.061** | **-1.158** | **-4.904** | | **A_TYR84** | | **-17.490** | **-6.416** | | | **-11.074** |
| **A_LYS83** | **56.453** | **-6.670** | | **63.123** | **A_LYS83** | **85.213** | **-7.527** | | **92.740** | **A_GLY85** | | **-12.242** | **-1.611** | **-10.631** | | **A_GLY85** | | **-22.438** | **-3.923** | | | **-18.515** |
| **A_TYR84** | **-8.798** | **3.380** | | **-12.178** | **A_TYR84** | **-37.739** | **0.740** | | **-38.479** | **A_ASP86** | | **-49.328** | **-4.513** | **-44.815** | | **A_ASP86** | | **-163.240** | **-0.151** | | | **-163.089** |
| **A_GLY85** | **-0.409** | **9.476** | | **-9.885** | **A_GLY85** | **-25.500** | **-0.542** | | **-24.958** | A_GLY87 | | -6.435 | -0.392 | -6.043 | | **A_GLY87** | | **-6.230** | **-2.279** | | | **-3.951** |
| **A_ASP86** | **-120.990** | **4.523** | | **-125.513** | **A_ASP86** | **-104.861** | **-0.841** | | **-104.020** | **A_SER88** | | **-21.235** | **-1.216** | **-20.018** | | **A_SER88** | | **-17.160** | **-3.588** | | | **-13.572** |
| **A_GLY87** | **-2.087** | **4.304** | | **-6.391** | **A_GLY87** | **0.500** | **-0.709** | | **1.208** | **A_TYR89** | | **-6.120** | **-2.352** | **-3.768** | | A_TYR89 | | -10.613 | -0.368 | | | -10.244 |
| **A_SER88** | **-5.499** | **-0.330** | | **-5.169** | **A_SER88** | **-22.285** | **-1.380** | | **-20.904** | **A_ALA90** | | **-18.349** | **-0.883** | **-17.466** | | A_ALA90 | | -8.939 | -0.104 | | | -8.835 |
| **A_SER118** | **-29.312** | **-1.178** | | **-28.135** | A_SER118 | -25.197 | -0.353 | | -24.843 | **A_ALA119** | | **1.801** | **-2.994** | **4.795** | | **A_ALA119** | | **7.274** | **-2.105** | | | **9.379** |
| **A_ALA119** | **13.351** | **-1.839** | | **15.190** | A_ALA119 | -7.954 | -1.229 | | -6.725 | **A_ARG120** | | **-2.058** | **-3.298** | **1.240** | | **A_ARG120** | | **51.951** | **-4.744** | | | **56.695** |
| **A_ARG120** | **89.255** | **-5.250** | | **94.505** | **A_ARG120** | **70.169** | **-3.442** | | **73.611** | **A_ILE123** | | **-7.363** | **0.126** | **-7.489** | | **A_ILE123** | | **-0.928** | **-2.646** | | | **1.718** |
| **A_ILE123** | **2.888** | **-2.562** | | **5.450** | **A_ILE123** | **6.994** | **-2.862** | | **9.856** | **A_GLY131** | | **3.632** | **-2.160** | **5.792** | | **A_GLY131** | | **-21.426** | **-2.546** | | | **-18.880** |
| A_LEU124 | -33.004 | -0.512 | | -32.493 | **A_LEU124** | **-28.523** | **-0.057** | | **-28.466** | **A_GLU132** | | **-16.737** | **-2.318** | **-14.419** | | **A_GLU132** | | **0.284** | **-0.899** | | | **1.183** |
| **A_PHE128** | **-20.240** | **-1.609** | | **-18.631** | A_PHE128 | -22.197 | -0.319 | | -21.878 | **A_ALA133** | | **-11.062** | **1.159** | **-12.221** | | **A_ALA133** | | **0.768** | **-1.829** | | | **2.598** |
| **A_SER130** | **-48.985** | **-0.851** | | **-48.134** | **A_SER130** | **-12.020** | **-1.010** | | **-11.010** | A_THR134A | | -10.666 | -0.384 | -10.281 | | **A_THR134A** | | **-18.722** | **-1.330** | | | **-17.392** |
| A_GLY131 | -18.283 | -1.733 | | -16.550 | **A_GLY131** | **-15.376** | **-3.117** | | **-12.259** | A_GLU134 | | -31.877 | -0.062 | -31.814 | | **A_GLU134** | | **-105.411** | **0.656** | | | **-106.067** |
| A_GLU132 | -9.084 | -0.881 | | -8.203 | **A_GLU132** | **2.826** | **-1.808** | | **4.634** | **A_LYS192** | | **0.484** | **-2.947** | **3.431** | | **A_LYS192** | | **49.329** | **-4.199** | | | **53.528** |
| **A_ALA133** | **-7.301** | **-3.163** | | **-4.137** | **A_ALA133** | **-4.894** | **-2.475** | | **-2.420** | **A_LYS193** | | **23.804** | **-6.502** | **30.306** | | **A_LYS193** | | **120.797** | **-5.778** | | | **126.575** |
| **A_SER190** | **-9.980** | **-1.813** | | **-8.167** | A_SER190 | -14.426 | -0.076 | | -14.350 | **A_LEU194** | | **-3.565** | **-0.390** | **-3.175** | | A_LEU194 | | -1.366 | -0.284 | | | -1.082 |
| **A_GLU191** | **-86.216** | **0.215** | | **-86.431** | A_GLU191 | -4.616 | -0.228 | | -4.388 | **A_LEU216** | | **-3.540** | **-1.449** | **-2.091** | | **A_LEU216** | | **-4.056** | **-1.812** | | | **-2.244** |
| **A_LYS192** | **132.075** | **2.238** | | **129.837** | **A_LYS192** | **-12.750** | **-2.513** | | **-10.238** | **A_ASP218** | | **-112.585** | **-0.849** | **-111.736** | | **A_ASP218** | | **-152.327** | **4.019** | | | **-156.346** |
| **A_LYS193** | **76.518** | **-5.330** | | **81.848** | **A_LYS193** | **13.865** | **-4.530** | | **18.395** | **A_SER219** | | **-28.138** | **-2.412** | **-25.726** | | A_SER219 | | -5.537 | -0.560 | | | -4.977 |
| **A_LEU194** | **-15.339** | **-3.278** | | **-12.061** | A_LEU194 | -2.793 | -0.151 | | -2.642 | **A_GLY220** | | **10.301** | **-1.818** | **12.118** | | **A_GLY220** | | **-17.050** | **-4.830** | | | **-12.220** |
| **A_LEU216** | **-6.753** | **-3.827** | | **-2.926** | **A_LEU216** | **-1.923** | **-2.025** | | **0.101** | **A_THR221** | | **-9.459** | **-1.241** | **-8.218** | | **A_THR221** | | **-11.804** | **-5.292** | | | **-6.512** |
| **A_LEU217** | **-1.309** | **-0.982** | | **-0.327** | A_LEU217 | -9.535 | -0.060 | | -9.475 | **A_THR222** | | **-9.558** | **-1.833** | **-7.725** | | **A_THR222** | | **-28.392** | **-4.385** | | | **-24.007** |
| **A_ASP218** | **-182.025** | **0.915** | | **-182.940** | **A_ASP218** | **-77.703** | **-1.738** | | **-75.966** | A_ILE223 | | -18.522 | -0.864 | -17.659 | | **A_ILE223** | | **-20.747** | **-3.933** | | | **-16.814** |
| A_SER219 | -5.584 | -0.815 | | -4.768 | **A_SER219** | **-5.568** | **-0.653** | | **-4.915** | **A_TYR225** | | **-13.976** | **-1.453** | **-12.523** | | **A_TYR225** | | **-13.146** | **-3.788** | | | **-9.357** |
| **A_GLY220** | **-14.814** | **-2.162** | | **-12.652** | **A_GLY220** | **-9.186** | **-4.181** | | **-5.005** | **A_ASP245** | | **-8.471** | **-0.952** | **-7.519** | | A_ASP245 | | -2.696 | -0.001 | | | -2.695 |
| **A_THR221** | **-18.987** | **-4.269** | | **-14.718** | **A_THR221** | **0.749** | **-3.523** | | **4.272** | **A_VAL251** | | **-3.900** | **-1.369** | **-2.531** | | A_VAL251 | | -0.982 | -0.018 | | | -0.965 |
| **A_THR222** | **-6.196** | **0.945** | | **-7.141** | **A_THR222** | **-11.337** | **-5.901** | | **-5.437** | A_PHE281 | | -1.895 | -0.201 | -1.694 | | **A_PHE281** | | **-5.823** | **-5.134** | | | **-0.689** |
| **A_ILE223** | **-22.508** | **-2.104** | | **-20.404** | **A_ILE223** | **-15.177** | **-3.433** | | **-11.744** | A_THR283 | | -7.298 | -0.237 | -7.060 | | **A_THR283** | | **-7.101** | **-1.536** | | | **-5.565** |
| **A_TYR225** | **-14.663** | **-2.036** | | **-12.628** | **A_TYR225** | **-17.102** | **-1.405** | | **-15.697** | **A_GLU295** | | **-58.097** | **-1.279** | **-56.818** | | **A_GLU295** | | **-16.207** | **-0.972** | | | **-15.235** |
| **A_PHE281** | **-5.871** | **-0.055** | | **-5.816** | **A_PHE281** | **-9.764** | **-1.719** | | **-8.045** | A_ARG297 | | 22.857 | -0.620 | 23.477 | | **A_ARG297** | | **-8.924** | **-3.724** | | | **-5.200** |
| A_GLU295 | -46.560 | -0.075 | | -46.484 | **A_GLU295** | **-145.367** | **1.164** | | **-146.531** | A_ARG299 | | 9.212 | -0.745 | 9.957 | | **A_ARG299** | | **17.229** | **-0.729** | | | **17.958** |
| A_ARG297 | 51.293 | -0.571 | | 51.863 | **A_ARG297** | **92.941** | **-2.816** | | **95.757** | A_ASP303 | | -23.776 | -0.767 | -23.009 | | **A_ASP303** | | **-149.112** | **1.917** | | | **-151.029** |
| **A_ARG299** | **59.291** | **-1.476** | | **60.768** | **A_ARG299** | **54.552** | **0.459** | | **54.093** | A_ILE305 | | 3.242 | -0.808 | 4.050 | | **A_ILE305** | | **-8.959** | **-2.224** | | | **-6.735** |
| **A_ASP303** | **-91.193** | **-2.586** | | **-88.606** | **A_ASP303** | **-45.847** | **-1.960** | | **-43.887** | A_ARG312 | | 11.620 | -0.001 | 11.621 | | **A_ARG312** | | **-1.546** | **-0.849** | | | **-0.696** |
| **A_ILE305** | **-8.303** | **-2.647** | | **-5.656** | **A_ILE305** | **-3.937** | **-0.477** | | **-3.460** |  | |  |  |  | |  | |  |  | | |  |
| **A_ASP308** | **-87.934** | **-0.136** | | **-87.798** | **A_ASP308** | **-146.202** | **1.360** | | **-147.562** |  | |  |  |  | |  | |  |  | | |  |
| A_ARG312 | 3.957 | 0.000 | | 3.957 | **A_ARG312** | **71.865** | **-0.981** | | **72.846** |  | |  |  |  | |  | |  |  | | |  |
| Total within 3(A) | -853.348 | -51.006 | | -802.342 | Total within 3(A) | -991.526 | -88.396 | | -903.130 | Total within 3(A) | | -704.934 | -91.232 | | -613.702 | Total within 3(A) | | -805.292 | -101.741 | | | -703.551 |
| Total (IE) | -942.752 | -58.531 | | -884.221 | Total (IE) | -1110.904 | -92.650 | | -1018.254 | Total (IE) | | -816.216 | -96.684 | | -719.532 | Total (IE) | -1004.134 | | | -107.038 | | -897.096 |

| Residue  **TABLE S5:** Contribution of the interactions energy in kcal/mol of the exo-β-(1,3)-glucanases (PDB ID:1CZ1) in the binding residues within 3Å as highlighted in bold letter. | Interaction Energy (IE) | VDW | Electrostatic | | | Residue | Interaction Energy (IE) | VDW | Electrostatic | | Residue | Interaction Energy (IE) | | VDW | Electrostatic | | Residue | | Interaction Energy (IE) | VDW | | Electrostatic | |
| --- | --- | --- | --- | --- | --- | --- | --- | --- | --- | --- | --- | --- | --- | --- | --- | --- | --- | --- | --- | --- | --- | --- | --- |
| KABT-AMP GIWKKWIKKWLKKLLKKLWKKG-NH2 | | | | | | KU3 GIWKKWIKKWLKVLKNLF-NH2 | | | | | Uperin 3.6  GVIDAAKKVVNVLKNLF-NH2 | | | | | | Upn-Lys5  GVIKAAKKVVKVLKNLF-NH2 | | | | | | |
| A_VAL18 | 7.158 | -0.101 | | 7.259 | | **A_VAL18** | **-2.855** | **-3.918** | | **1.064** | **A_ASN19** | | **-16.503** | **-0.466** | **-16.037** | | A_ASN19 | | -13.896 | -1.056 | | | -12.840 |
| A_ASN19 | -12.143 | -0.455 | | -11.688 | | **A_ASN19** | **20.792** | **3.656** | | **17.136** | **A_GLU27** | | **-16.568** | **-2.581** | **-13.987** | | **A_GLU27** | | **-12.349** | **-3.079** | | | **-9.270** |
| A_LEU20 | -1.020 | -0.087 | | -0.932 | | **A_LEU20** | **2.170** | **-3.544** | | **5.715** | **A_TYR29** | | **-9.280** | **-1.971** | **-7.309** | | **A_TYR29** | | **-34.614** | **-4.409** | | | **-30.205** |
| **A_GLY21** | **1.814** | **-0.888** | | **2.702** | | **A_GLY21** | **-5.748** | **-1.687** | | **-4.062** | **A_MET30** | | **-5.956** | **-0.723** | **-5.233** | | **A_MET30** | | **-8.488** | **-1.800** | | | **-6.688** |
| **A_GLY22** | **-4.673** | **-0.819** | | **-3.854** | | **A_GLY22** | **-9.190** | **-0.513** | | **-8.676** | **A_TYR52** | | **2.626** | **0.265** | **2.362** | | A_TYR52 | | 1.595 | -0.575 | | | 2.171 |
| A_TRP23 | -4.395 | -0.091 | | -4.304 | | **A_TRP23** | **-5.162** | **0.886** | | **-6.048** | **A_HIS53** | | **9.045** | **-1.056** | **10.101** | | A_HIS53 | | -2.959 | -0.046 | | | -2.913 |
| A_PHE24 | -2.561 | -0.141 | | -2.420 | | **A_PHE24** | **-4.820** | **-0.956** | | **-3.865** | **A_ARG92** | | **149.957** | **-3.265** | **153.222** | | **A_ARG92** | | **27.122** | **-0.808** | | | **27.930** |
| **A_VAL25** | **-9.412** | **-2.089** | | **-7.323** | | **A_VAL25** | **-9.167** | **-2.297** | | **-6.870** | **A_PRO94** | | **-0.890** | **-0.854** | **-0.036** | | A_PRO94 | | 1.147 | -0.194 | | | 1.340 |
| **A_GLU27** | **-40.851** | **-2.057** | | **-38.794** | | **A_GLU27** | **-12.149** | **-3.240** | | **-8.909** | A_TYR97 | | -9.994 | -0.418 | -9.576 | | **A_TYR97** | | **-12.577** | **0.047** | | | **-12.624** |
| A_PRO28 | -2.367 | -0.369 | | -1.998 | | **A_PRO28** | **-6.349** | **-1.153** | | **-5.196** | A_TRP98 | | 5.437 | -0.146 | 5.583 | | **A_TRP98** | | **-3.973** | **-0.595** | | | **-3.377** |
| **A_TYR29** | **-28.512** | **1.475** | | **-29.987** | | **A_TYR29** | **-26.255** | **-2.255** | | **-24.000** | A_LEU103 | | 1.545 | -0.105 | 1.650 | | **A_LEU103** | | **2.000** | **-0.541** | | | **2.541** |
| **A_MET30** | **-0.635** | **0.636** | | **-1.272** | | **A_MET30** | **-2.765** | **-2.773** | | **0.008** | **A_TYR108** | | **-10.144** | **0.840** | **-10.984** | | **A_TYR108** | | **-9.659** | **-1.565** | | | **-8.094** |
| **A_GLU51** | **-12.278** | **-0.109** | | **-12.169** | | A_GLU51 | 5.363 | -0.078 | | 5.441 | A_LEU134 | | -4.263 | -0.580 | -3.683 | | **A_LEU134** | | **-13.389** | **-1.850** | | | **-11.539** |
| **A_TYR52** | **-0.819** | **-0.035** | | **-0.784** | | A_TYR52 | 1.293 | -0.003 | | 1.296 | **A_HIS135** | | **82.022** | **-3.179** | **85.202** | | **A_HIS135** | | **22.998** | **-4.485** | | | **27.483** |
| **A_THR55** | **0.423** | **-0.001** | | **0.424** | | A_THR55 | 2.866 | 0.000 | | 2.866 | **A_GLY136** | | **-7.802** | **-1.749** | **-6.053** | | **A_GLY136** | | **-7.576** | **-1.817** | | | **-5.758** |
| A_ILE75 | 0.228 | -0.004 | | 0.232 | | **A_ILE75** | **-3.576** | **-0.382** | | **-3.194** | **A_ALA137** | | **-10.298** | **-1.045** | **-9.253** | | **A_ALA137** | | **-11.638** | **-0.276** | | | **-11.361** |
| A_PHE80 | 0.000 | 0.000 | | 0.000 | | **A_PHE80** | **1.105** | **-2.936** | | **4.042** | **A_PRO138** | | **-5.813** | **-1.294** | **-4.519** | | **A_PRO138** | | **-6.979** | **-2.929** | | | **-4.049** |
| A_ILE83 | -1.241 | -0.001 | | -1.240 | | **A_ILE83** | **2.989** | **-1.501** | | **4.490** | **A_GLY139** | | **-5.761** | **-1.206** | **-4.555** | | **A_GLY139** | | **-5.040** | **0.391** | | | **-5.430** |
| A_PHE90 | -3.091 | -0.005 | | -3.086 | | **A_PHE90** | **2.798** | **-2.972** | | **5.770** | **A_SER140** | | **-24.154** | **4.398** | **-28.552** | | **A_SER140** | | **5.710** | **-0.130** | | | **5.840** |
| A_VAL91 | 1.608 | -0.001 | | 1.608 | | **A_VAL91** | **2.040** | **4.937** | | **-2.897** | **A_GLN141** | | **0.380** | **1.415** | **-1.035** | | **A_GLN141** | | **1.732** | **2.106** | | | **-0.374** |
| A_ARG92 | 87.207 | -1.102 | | 88.309 | | **A_ARG92** | **18.687** | **0.478** | | **18.209** | **A_ASN142** | | **-10.044** | **0.588** | **-10.632** | | **A_ASN142** | | **-13.606** | **-3.569** | | | **-10.036** |
| A_ILE93 | -4.442 | -0.050 | | -4.391 | | **A_ILE93** | **0.324** | **-0.594** | | **0.917** | **A_GLY143** | | **-11.385** | **-0.652** | **-10.733** | | **A_GLY143** | | **-3.304** | **-2.081** | | | **-1.223** |
| **A_PRO94** | **-0.061** | **-0.942** | | **0.881** | | **A_PRO94** | **-11.580** | **-1.112** | | **-10.468** | **A_PHE144** | | **-18.979** | **-2.148** | **-16.831** | | **A_PHE144** | | **-5.714** | **-2.598** | | | **-3.116** |
| **A_TYR97** | **-9.320** | **-0.198** | | **-9.122** | | A_TYR97 | 1.095 | -0.022 | | 1.117 | **A_ASP145** | | **-39.256** | **-0.499** | **-38.757** | | **A_ASP145** | | **-28.359** | **1.148** | | | **-29.507** |
| **A_TRP98** | **5.578** | **-0.381** | | **5.959** | | A_TRP98 | -1.660 | -0.121 | | -1.538 | **A_ASN146** | | **-2.428** | **1.798** | **-4.226** | | **A_ASN146** | | **-8.670** | **-0.326** | | | **-8.344** |
| **A_LEU103** | **-0.089** | **-0.001** | | **-0.088** | | A_LEU103 | 0.110 | 0.000 | | 0.110 | **A_SER147** | | **-21.494** | **-5.666** | **-15.828** | | **A_SER147** | | **4.622** | **4.801** | | | **-0.179** |
| **A_TYR108** | **-0.260** | **-0.030** | | **-0.230** | | A_TYR108 | -6.831 | -0.031 | | -6.800 | **A_ARG150** | | **-1.276** | **-1.917** | **0.640** | | **A_ARG150** | | **-10.195** | **-0.209** | | | **-9.986** |
| A_ALA119 | 0.000 | 0.000 | | 0.000 | | **A_ALA119** | **-2.981** | **-1.283** | | **-1.699** | **A_ASP151** | | **-28.831** | **1.864** | **-30.694** | | **A_ASP151** | | **-37.440** | **0.813** | | | **-38.253** |
| A_LEU120 | 0.040 | 0.000 | | 0.040 | | **A_LEU120** | **-5.978** | **-1.847** | | **-4.131** | **A_SER152** | | **-15.704** | **-3.447** | **-12.257** | | **A_SER152** | | **-11.853** | **-1.239** | | | **-10.613** |
| A_VAL130 | 3.185 | 0.000 | | 3.185 | | **A_VAL130** | **7.744** | **-0.950** | | **8.694** | **A_TYR153** | | **-20.629** | **-3.586** | **-17.043** | | A_TYR153 | | -13.735 | -0.853 | | | -12.881 |
| **A_ASP133** | **-123.655** | **-1.314** | | **-122.341** | | **A_ASP133** | **-38.162** | **-0.967** | | **-37.195** | **A_GLU188** | | **-24.716** | **-1.498** | **-23.218** | | A_GLU188 | | -16.590 | -0.092 | | | -16.499 |
| **A_LEU134** | **-17.467** | **0.703** | | **-18.170** | | A_LEU134 | -1.378 | -0.217 | | -1.161 | **A_LEU190** | | **-7.016** | **-1.737** | **-5.280** | | A_LEU190 | | -7.194 | -0.948 | | | -6.247 |
| **A_HIS135** | **91.314** | **-3.332** | | **94.646** | | **A_HIS135** | **29.638** | **-1.005** | | **30.643** | **A_ASN191** | | **-27.335** | **0.108** | **-27.443** | | **A_ASN191** | | **-19.158** | **-1.140** | | | **-18.017** |
| **A_GLY136** | **-18.883** | **-2.538** | | **-16.346** | | **A_GLY136** | **-4.229** | **-0.712** | | **-3.517** | **A_GLU192** | | **-174.610** | **0.546** | **-175.156** | | **A_GLU192** | | **-158.074** | **-3.761** | | | **-154.313** |
| **A_ALA137** | **-0.213** | **-0.783** | | **0.570** | | A_ALA137 | 0.353 | -0.169 | | 0.522 | **A_PRO193** | | **-19.591** | **-2.986** | **-16.605** | | **A_PRO193** | | **-18.979** | **1.498** | | | **-20.477** |
| **A_PRO138** | **-14.091** | **-0.134** | | **-13.957** | | A_PRO138 | 0.561 | -0.017 | | 0.578 | **A_LEU198** | | **-1.937** | **-0.955** | **-0.982** | | **A_LEU194** | | **-10.396** | **-2.449** | | | **-7.947** |
| **A_GLY139** | **-2.848** | **-0.123** | | **-2.726** | | A_GLY139 | 1.597 | -0.012 | | 1.609 | A_ILE225 | | -6.146 | -0.040 | -6.106 | | **A_LEU198** | | **-0.599** | **-1.457** | | | **0.858** |
| **A_SER140** | **-6.889** | **-1.013** | | **-5.876** | | A_SER140 | 0.468 | -0.168 | | 0.636 | **A_HIS226** | | **92.097** | **-2.013** | **94.111** | | A_ILE225 | | 0.598 | -0.002 | | | 0.600 |
| **A_GLN141** | **-25.586** | **0.037** | | **-25.623** | | **A_GLN141** | **-2.738** | **-1.178** | | **-1.560** | **A_PHE229** | | **-3.710** | **-2.273** | **-1.437** | | **A_PHE229** | | **-29.298** | **-1.426** | | | **-27.871** |
| **A_ASN142** | **19.047** | **9.047** | | **10.000** | | **A_ASN142** | **-13.409** | **2.490** | | **-15.899** | **A_ASP251** | | **-148.171** | **0.038** | **-148.209** | | A_ASP251 | | -37.859 | -0.225 | | | -37.634 |
| **A_GLY143** | **-11.325** | **-1.098** | | **-10.227** | | **A_GLY143** | **-9.043** | **0.148** | | **-9.190** | **A_HIS253** | | **89.253** | **-3.928** | **93.180** | | **A_HIS253** | | **19.661** | **-0.007** | | | **19.668** |
| **A_PHE144** | **-18.406** | **-6.195** | | **-12.211** | | **A_PHE144** | **-4.419** | **-3.383** | | **-1.037** | **A_HIS254** | | **11.666** | **-1.418** | **13.084** | | **A_HIS254** | | **28.235** | **-2.297** | | | **30.532** |
| **A_ASP145** | **-73.598** | **-0.456** | | **-73.142** | | **A_ASP145** | **-126.582** | **7.856** | | **-134.438** | **A_TYR255** | | **-33.463** | **-3.523** | **-29.940** | | **A_TYR255** | | **-21.291** | **-4.812** | | | **-16.479** |
| **A_ASN146** | **-26.598** | **-9.279** | | **-17.319** | | **A_ASN146** | **-5.769** | **0.661** | | **-6.430** | **A_PHE258** | | **-10.001** | **-3.930** | **-6.071** | | **A_PHE258** | | **-11.735** | **-2.318** | | | **-9.417** |
| **A_SER147** | **-6.835** | **-1.430** | | **-5.404** | | **A_SER147** | **3.949** | **-0.623** | | **4.573** | **A_GLU292** | | **-21.856** | **-7.775** | **-14.081** | | **A_GLU292** | | **-9.232** | **-6.866** | | | **-2.366** |
| **A_GLY148** | **0.087** | **-1.049** | | **1.137** | | A_GLY148 | -7.834 | -0.255 | | -7.578 | **A_TRP293** | | **-11.429** | **0.616** | **-12.045** | | **A_TRP293** | | **-17.086** | **-4.316** | | | **-12.770** |
| **A_LEU149** | **-6.758** | **-0.696** | | **-6.062** | | A_LEU149 | 0.143 | -0.110 | | 0.253 | **A_SER294** | | **-4.567** | **0.308** | **-4.875** | | **A_SER294** | | **-6.810** | **-1.040** | | | **-5.770** |
| **A_ARG150** | **1.222** | **-0.963** | | **2.185** | | A_ARG150 | 65.862 | -0.572 | | 66.434 | **A_ALA295** | | **-14.579** | **-3.892** | **-10.687** | | **A_ALA295** | | **-7.470** | **2.016** | | | **-9.485** |
| **A_ASP151** | **-47.998** | **-0.983** | | **-47.015** | | A_ASP151 | -78.334 | -0.578 | | -77.756 | **A_ALA296** | | **-17.341** | **-1.791** | **-15.550** | | **A_ALA296** | | **-4.101** | **-0.668** | | | **-3.433** |
| **A_SER152** | **-4.489** | **-1.750** | | **-2.739** | | A_SER152 | -6.383 | -0.064 | | -6.318 | **A_LEU304** | | **-8.188** | **-3.221** | **-4.968** | | A_LEU304 | | -10.746 | -1.504 | | | -9.242 |
| **A_TYR153** | **-12.612** | **-1.323** | | **-11.289** | | **A_TYR153** | **-11.681** | **-0.854** | | **-10.827** | **A_ILE332** | | **-2.553** | **-2.062** | **-0.491** | | **A_ILE332** | | **-0.315** | **-1.330** | | | **1.015** |
| **A_GLU188** | **-9.505** | **-0.908** | | **-8.596** | | A_GLU188 | -18.143 | -0.135 | | -18.007 | **A_TRP335** | | **-4.974** | **-1.954** | **-3.020** | | **A_TRP335** | | **-0.191** | **-0.325** | | | **0.133** |
| **A_LEU189** | **-17.569** | **-0.790** | | **-16.779** | | A_LEU189 | -0.989 | -0.010 | | -0.980 | A_HIS339 | | 20.775 | -0.091 | 20.866 | | **A_HIS339** | | **48.158** | **-0.556** | | | **48.714** |
| **A_LEU190** | **-4.607** | **-1.586** | | **-3.021** | | A_LEU190 | 0.071 | -0.256 | | 0.327 | **A_LEU351** | | **-1.609** | **-1.158** | **-0.450** | | A_LEU351 | | 2.568 | -0.037 | | | 2.605 |
| **A_ASN191** | **-13.056** | **-6.807** | | **-6.249** | | **A_ASN191** | **-23.363** | **-4.884** | | **-18.479** | **A_TRP360** | | **-8.829** | **-2.856** | **-5.974** | | A_TRP360 | | -14.338 | -0.119 | | | -14.219 |
| **A_GLU192** | **-190.710** | **-2.170** | | **-188.540** | | **A_GLU192** | **-69.024** | **0.667** | | **-69.690** | **A_VAL361** | | **-8.493** | **-2.393** | **-6.100** | | A_VAL361 | | 0.647 | -0.471 | | | 1.118 |
| **A_LEU194** | **-1.692** | **0.556** | | **-2.248** | | **A_LEU194** | **-2.648** | **-0.813** | | **-1.836** | **A_PHE362** | | **-8.075** | **-3.974** | **-4.101** | | **A_PHE362** | | **-18.572** | **4.293** | | | **-22.865** |
| **A_VAL197** | **-0.438** | **-0.567** | | **0.128** | | A_VAL197 | 0.476 | -0.402 | | 0.878 | **A_TRP363** | | **-21.966** | **-5.389** | **-16.577** | | **A_TRP363** | | **-15.530** | **4.802** | | | **-20.332** |
| **A_PHE229** | **-8.738** | **-2.686** | | **-6.052** | | **A_PHE229** | **-16.631** | **-6.699** | | **-9.931** | **A_SER364** | | **-32.487** | **-1.461** | **-31.026** | | **A_SER364** | | **-34.458** | **-1.225** | | | **-33.233** |
| **A_HIS252** | **14.916** | **-0.655** | | **15.571** | | A_HIS252 | 50.618 | -0.313 | | 50.931 | **A_TRP365** | | **-5.649** | **-1.186** | **-4.463** | | A_TRP365 | | -13.887 | -0.698 | | | -13.189 |
| **A_HIS253** | **74.341** | **-1.114** | | **75.455** | | **A_HIS253** | **56.727** | **-0.371** | | **57.098** | A_LYS366 | | -13.012 | -0.233 | -12.779 | | **A_LYS366** | | **3.573** | **-1.191** | | | **4.764** |
| **A_HIS254** | **22.609** | **-3.101** | | **25.710** | | **A_HIS254** | **79.079** | **5.787** | | **73.293** | A_THR367 | | -11.391 | -0.243 | -11.148 | | **A_THR367** | | **-17.030** | **-2.618** | | | **-14.412** |
| **A_GLN256** | **-32.962** | **-4.214** | | **-28.748** | | A_GLN256 | 13.925 | -0.335 | | 14.260 | A_ALA370 | | -6.901 | -0.231 | -6.670 | | **A_ALA370** | | **-7.775** | **-1.836** | | | **-5.940** |
| **A_VAL257** | **-25.742** | **-1.484** | | **-24.258** | | A_VAL257 | -2.327 | -0.582 | | -1.745 | **A_PRO371** | | **-22.073** | **1.174** | **-23.247** | | **A_PRO371** | | **2.836** | **2.631** | | | **0.205** |
| **A_PHE258** | **-5.161** | **-4.001** | | **-1.160** | | **A_PHE258** | **-24.192** | **-7.070** | | **-17.123** | **A_TRP373** | | **-4.154** | **-4.349** | **0.196** | | **A_TRP373** | | **2.245** | **17.100** | | | **-14.855** |
| A_SER259 | -13.862 | -0.053 | | -13.809 | | **A_SER259** | **-13.280** | **-0.841** | | **-12.439** | **A_SER374** | | **-18.602** | **0.358** | **-18.960** | | **A_SER374** | | **-1.342** | **6.902** | | | **-8.244** |
| **A_LEU263** | **-17.566** | **-1.205** | | **-16.360** | | A_LEU263 | -1.891 | -0.127 | | -1.764 | **A_PHE375** | | **-9.818** | **-0.006** | **-9.811** | | **A_PHE375** | | **2.990** | **1.387** | | | **1.602** |
| **A_ARG265** | **-4.619** | **-0.558** | | **-4.061** | | A_ARG265 | 49.068 | -0.025 | | 49.093 | **A_THR377** | | **0.700** | **-0.630** | **1.330** | | **A_THR377** | | **-4.442** | **-1.816** | | | **-2.626** |
| **A_ILE267** | **-6.304** | **-1.356** | | **-4.948** | | A_ILE267 | 0.000 | 0.000 | | 0.000 | **A_LEU378** | | **-1.309** | **-2.249** | **0.941** | | **A_LEU378** | | **-7.541** | **-3.231** | | | **-4.310** |
| **A_HIS270** | **66.494** | **-4.457** | | **70.951** | | A_HIS270 | -0.708 | 0.000 | | -0.708 | **A_PHE384** | | **-3.513** | **-3.027** | **-0.486** | | A_PHE384 | | -7.573 | -1.339 | | | -6.234 |
| **A_ALA274** | **-1.495** | **-0.477** | | **-1.018** | | A_ALA274 | -5.338 | -0.002 | | -5.336 |  | |  |  |  | |  | |  |  | | |  |
| **A_TRP277** | **-0.831** | **-0.926** | | **0.095** | | A_TRP277 | -3.112 | -0.371 | | -2.741 |  | |  |  |  | |  | |  |  | | |  |
| **A_ALA290** | **0.390** | **-1.399** | | **1.790** | | A_ALA290 | -6.616 | -0.127 | | -6.489 |  | |  |  |  | |  | |  |  | | |  |
| **A_GLU292** | **-23.647** | **0.750** | | **-24.397** | | **A_GLY291** | **-22.716** | **-1.920** | | **-20.796** |  | |  |  |  | |  | |  |  | | |  |
| **A_TRP293** | **-1.283** | **1.313** | | **-2.596** | | **A_GLU292** | **-28.475** | **-6.299** | | **-22.176** |  | |  |  |  | |  | |  |  | | |  |
| **A_SER294** | **-45.525** | **-0.110** | | **-45.415** | | **A_TRP293** | **-25.636** | **-0.723** | | **-24.913** |  | |  |  |  | |  | |  |  | | |  |
| **A_ALA295** | **32.722** | **31.225** | | **1.498** | | **A_SER294** | **-10.061** | **-0.804** | | **-9.257** |  | |  |  |  | |  | |  |  | | |  |
| **A_ALA296** | **16.087** | **20.534** | | **-4.447** | | **A_ALA295** | **-7.891** | **-0.362** | | **-7.529** |  | |  |  |  | |  | |  |  | | |  |
| **A_LEU297** | **-16.758** | **-5.050** | | **-11.708** | | A_ALA296 | -4.963 | -0.158 | | -4.806 |  | |  |  |  | |  | |  |  | | |  |
| **A_ASP299** | **-173.175** | **-1.541** | | **-171.634** | | A_LEU297 | 1.579 | -0.012 | | 1.591 |  | |  |  |  | |  | |  |  | | |  |
| A_CYS300 | -10.112 | -0.422 | | -9.690 | | **A_ASP299** | **-103.987** | **-0.555** | | **-103.432** |  | |  |  |  | |  | |  |  | | |  |
| A_ALA301 | 0.159 | -0.554 | | 0.713 | | **A_CYS300** | **-30.001** | **0.245** | | **-30.246** |  | |  |  |  | |  | |  |  | | |  |
| **A_LEU304** | **-19.890** | **0.110** | | **-20.000** | | **A_ALA301** | **-30.290** | **-0.639** | | **-29.651** |  | |  |  |  | |  | |  |  | | |  |
| **A_ASN305** | **-41.093** | **-3.268** | | **-37.825** | | **A_LEU304** | **-3.627** | **-0.952** | | **-2.675** |  | |  |  |  | |  | |  |  | | |  |
| A_ARG309 | 38.398 | -0.151 | | 38.549 | | **A_ASN305** | **-18.775** | **-6.262** | | **-12.513** |  | |  |  |  | |  | |  |  | | |  |
| A_GLY310 | -1.209 | -0.075 | | -1.134 | | **A_ARG309** | **82.012** | **-4.089** | | **86.101** |  | |  |  |  | |  | |  |  | | |  |
| A_ALA311 | 5.657 | -0.286 | | 5.943 | | **A_GLY310** | **-25.316** | **-1.265** | | **-24.051** |  | |  |  |  | |  | |  |  | | |  |
| **A_ARG312** | **72.388** | **-0.523** | | **72.911** | | **A_ALA311** | **-6.345** | **-1.142** | | **-5.203** |  | |  |  |  | |  | |  |  | | |  |
| **A_TYR313** | **-18.641** | **-1.057** | | **-17.584** | | **A_ARG312** | **123.372** | **-1.625** | | **124.997** |  | |  |  |  | |  | |  |  | | |  |
| A_TYR317 | 0.342 | -0.111 | | 0.453 | | A_TYR313 | 2.806 | -0.078 | | 2.884 |  | |  |  |  | |  | |  |  | | |  |
| A_ASP318 | -35.206 | -0.017 | | -35.189 | | **A_TYR317** | **-28.415** | **-1.433** | | **-26.981** |  | |  |  |  | |  | |  |  | | |  |
| **A_LEU329** | **-29.135** | **0.067** | | **-29.202** | | **A_ASP318** | **-151.207** | **-1.535** | | **-149.672** |  | |  |  |  | |  | |  |  | | |  |
| **A_LEU330** | **-27.366** | **-0.874** | | **-26.492** | | A_LEU329 | -1.707 | -0.003 | | -1.704 |  | |  |  |  | |  | |  |  | | |  |
| **A_ASP331** | **-66.244** | **-0.983** | | **-65.261** | | A_LEU330 | -2.559 | -0.011 | | -2.549 |  | |  |  |  | |  | |  |  | | |  |
| **A_ILE332** | **-4.887** | **-1.442** | | **-3.445** | | A_ASP331 | -0.226 | 0.000 | | -0.226 |  | |  |  |  | |  | |  |  | | |  |
| **A_TRP335** | **3.650** | **-3.091** | | **6.741** | | A_ILE332 | 0.372 | 0.000 | | 0.372 |  | |  |  |  | |  | |  |  | | |  |
| **A_ASP342** | **-101.758** | **-2.116** | | **-99.642** | | A_TRP335 | 2.798 | -0.001 | | 2.800 |  | |  |  |  | |  | |  |  | | |  |
| **A_THR343** | **-7.064** | **4.109** | | **-11.173** | | A_ASP342 | 0.000 | 0.000 | | 0.000 |  | |  |  |  | |  | |  |  | | |  |
| **A_ARG344** | **42.489** | **-3.092** | | **45.581** | | A_THR343 | -4.621 | -0.003 | | -4.618 |  | |  |  |  | |  | |  |  | | |  |
| **A_TYR346** | **-22.643** | **-3.303** | | **-19.340** | | A_ARG344 | -0.768 | 0.000 | | -0.768 |  | |  |  |  | |  | |  |  | | |  |
| **A_ILE347** | **4.296** | **0.776** | | **3.520** | | A_TYR346 | -10.427 | -0.028 | | -10.399 |  | |  |  |  | |  | |  |  | | |  |
| **A_GLN350** | **-0.890** | **-0.476** | | **-0.414** | | A_GLN350 | -10.892 | -0.072 | | -10.820 |  | |  |  |  | |  | |  |  | | |  |
| **A_PHE354** | **1.134** | **-0.272** | | **1.405** | | A_PHE354 | 0.709 | -0.054 | | 0.764 |  | |  |  |  | |  | |  |  | | |  |
| A_VAL361 | -14.002 | -0.438 | | -13.564 | | **A_VAL361** | **-7.140** | **-2.337** | | **-4.803** |  | |  |  |  | |  | |  |  | | |  |
| **A_PHE362** | **-2.846** | **-2.434** | | **-0.412** | | **A_PHE362** | **-7.601** | **-1.613** | | **-5.988** |  | |  |  |  | |  | |  |  | | |  |
| **A_TRP363** | **-26.475** | **-7.255** | | **-19.221** | | **A_TRP363** | **-15.535** | **4.355** | | **-19.891** |  | |  |  |  | |  | |  |  | | |  |
| **A_SER364** | **-22.371** | **-0.392** | | **-21.979** | | A_SER364 | -16.847 | -1.319 | | -15.529 |  | |  |  |  | |  | |  |  | | |  |
| A_TRP365 | -2.885 | -0.242 | | -2.643 | | **A_TRP365** | **-7.719** | **-1.778** | | **-5.941** |  | |  |  |  | |  | |  |  | | |  |
| **A_PRO371** | **-8.813** | **-2.052** | | **-6.760** | | A_PRO371 | -6.035 | -0.022 | | -6.013 |  | |  |  |  | |  | |  |  | | |  |
| **A_GLU372** | **-166.087** | **-6.794** | | **-159.293** | | A_GLU372 | -71.101 | -0.368 | | -70.733 |  | |  |  |  | |  | |  |  | | |  |
| **A_TRP373** | **553.449** | **562.962** | | **-9.513** | | **A_TRP373** | **-15.311** | **-0.688** | | **-14.624** |  | |  |  |  | |  | |  |  | | |  |
| **A_PHE375** | **-2.746** | **-0.755** | | **-1.991** | | A_PHE375 | -8.892 | -0.146 | | -8.747 |  | |  |  |  | |  | |  |  | | |  |
| **A_LEU383** | **-22.025** | **-1.616** | | **-20.409** | | A_LEU383 | -3.653 | 0.000 | | -3.653 |  | |  |  |  | |  | |  |  | | |  |
| Total within 3(A) | -141.763 | 1058.559 | | | -1200.322 | Total within 3(A) | -586.364 | -69.166 | | -517.198 | Total within 3(A) | | -618.518 | -97.053 | | -521.465 | Total within 3(A) | | -561.626 | -19.811 | | | -541.815 |
| Total (IE) | -657.621 | 498.575 | | -1156.196 | | Total (IE) | -668.467 | -76.545 | | -591.922 | Total (IE) | | -552.011 | -98.706 | | -453.305 | Total (IE) | -627.185 | | | -39.221 | | -587.965 |
